# Large-scale multi-omics analysis suggests specific roles for intragenic cohesin in transcriptional regulation

**DOI:** 10.1038/s41467-022-30792-9

**Published:** 2022-06-09

**Authors:** Jiankang Wang, Masashige Bando, Katsuhiko Shirahige, Ryuichiro Nakato

**Affiliations:** 1 Institute for Quantitative Biosciences, The University of Tokyo, Tokyo, Japan; 2 Graduate School of Medicine, The University of Tokyo, Tokyo, Japan; 3 Department of Biosciences and Nutrition, Karolinska Institutet, Stockholm, Sweden

**Keywords:** Computational biology and bioinformatics, Epigenomics, Chromatin structure, Transcription, Diseases

## Abstract

Cohesin, an essential protein complex for chromosome segregation, regulates transcription through a variety of mechanisms. It is not a trivial task to assign diverse cohesin functions. Moreover, the context-specific roles of cohesin-mediated interactions, especially on intragenic regions, have not been thoroughly investigated. Here we perform a comprehensive characterization of cohesin binding sites in several human cell types. We integrate epigenomic, transcriptomic and chromatin interaction data to explore the context-specific functions of intragenic cohesin related to gene activation. We identify a specific subset of cohesin binding sites, decreased intragenic cohesin sites (DICs), which are negatively correlated with transcriptional regulation. A subgroup of DICs is enriched with enhancer markers and RNA polymerase II, while the others are more correlated to chromatin architecture. DICs are observed in various cell types, including cells from patients with cohesinopathy. We also implement machine learning to our data and identified genomic features for isolating DICs from all cohesin sites. These results suggest a previously unidentified function of cohesin on intragenic regions for transcriptional regulation.

## Abstract (web summary)

Cohesin complex is essential for transcriptional regulation and chromosome folding. Here the authors provide a large-scale multi-omics analysis suggesting that cohesin bound on gene bodies has a specific role in transcriptional regulation.

## Introduction

Cohesin, a ring-shaped chromosome-bound protein complex, is required for holding sister chromatids together during certain phases of the cell cycle^1^. Recent studies suggest that cohesin also has a role in transcriptional regulation, maintenance of chromosome architecture^2^ and DNA repair^3^. Context-specific functions of cohesin have been investigated using chromatin immunoprecipitation followed by sequencing (ChIP-seq) and high-throughput chromosome conformation capture (Hi-C). The early study reported that most cohesin-binding sites overlap with CTCF to function as an insulator^4^. Conversely, a group of cohesin has been reported to be CTCF independent and co-bind with tissue-specific transcription factors (TFs) to contribute to transcriptional regulation^5,6^, possibly via mediating interactions between enhancers and promoters^7^. Other studies using Hi-C have shown that cohesin and CTCF are essential for the formation of topologically associated domains (TADs), evolutionarily conserved chromatin domains ranging from a few hundred kilobases to several megabases in length^8,9^. These studies focused on cohesin functions with respect to insulation, or the formation of enhancer-promoter interactions that implicitly assume the positive regulation of gene expression. In contrast, a recent report showed that transcription elongation within gene bodies causes displacement of cohesin binding from chromatin, leading to disruption of cohesin-mediated loops^10^. Thus, a subset of chromatin loops (either end of which may be located on intragenic regions) mediated by cohesin is suggested to be negatively correlated with gene activation. While modifications in intragenic regions affect transcriptional events^11–13^, the function of intragenic cohesin has hardly been discussed.

Mutations in the cohesin complex and its loader (NIPBL) are observed in the cohesinopathy Cornelia de Lange syndrome (CdLS), a multisystem developmental disorder^14^, and in multiple types of cancers^15,16^. Our previous study found that the diagnostic phenotype of CdLS is very similar to that of CHOPS syndrome^17^, which is caused by missense mutations in AFF4, a core component of the super elongation complex. Given the diverse functions of cohesin in gene expression and chromatin folding, the underlying molecular mechanism responsible for the similarity between CdLS and CHOPS is yet unknown. Noteworthily, the CHOPS-related mutations in the super elongation complex are also associated with transcriptional regulation by cohesin, indicating a common pathogenetic mechanism of cohesin in CHOPS and CdLS. It could be a feasible hypothesis that intragenic cohesin has a distinct role that links the phenotypic similarity between CdLS and CHOPS.

Here, we conducted a large-scale epigenomic analysis to clarify the context-specific functions of cohesin sites, especially in intragenic regions. To investigate the perturbation of cohesin binding sites by gene activation, we generated RNA sequencing (RNA-seq) and ChIP-seq data for cohesin and several TFs in MCF-7 cells with or without transcription stimulus. We also used many publicly available datasets, including Hi-C, ChIP-seq, RNA-seq and chromatin interaction analysis by paired-end tag (ChIA-PET). First, we clarified that a subset of cohesin sites, which we refer to as ‘decreased intragenic cohesin sites’ (DICs), is distinct from the other groups of cohesin sites. Cohesin binding on DICs is negatively correlated with transcriptional activation and locus compaction of chromatin. A part of DICs exhibit a high preference for enhancer marks and paused RNA polymerase II, whereas others contribute to chromatin architecture. Second, we performed ChIP-seq and RNA-seq with cohesin-depleted cells and suggested that cohesin has an active function on DICs. Third, we applied machine learning and captured DICs with a distinct epigenomic landscape, which is predictable across cell types. Finally, we conducted plenty of ChIP-seq in other cell types. Importantly, DICs can be observed across multiple cell types, including cells derived from CdLS and CHOPS patients, in a cell-type-specific manner. The findings from our integrated analysis and machine learning approaches suggest an additional role for cohesin in the regulation of gene expression.

## Results

### Classification of DICs

**Fig. 1:**
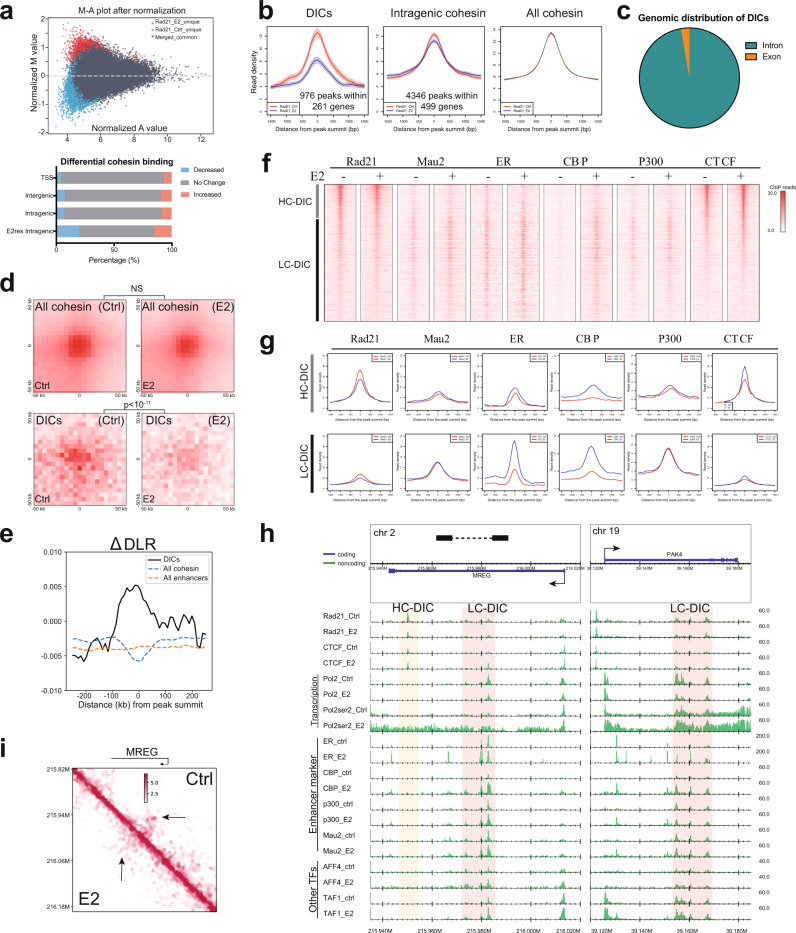
Classification of decreased intragenic cohesin (DIC) sites. **a** Quantitative comparison of cohesin peak intensity between control (Ctrl) and estrogen (E2)-treated MCF-7 cells. M value is the log_2_(fold change) of normalized read densities under comparison. A value is the average signal strength of each peak. The lower panel shows the proportion of cohesin binding that decreased or increased. **b** Average binding profiles on different cohesin sites. Shaded regions indicate 95% confidence intervals. Red, control. Blue, E2 treatment. **c** Genomic distribution of all DICs suggested most of DICs were located in introns. Source data are provided in the Source Data file. **d** Aggregate peak analysis around DICs (*p* = 3.5 × 10^−12^) or all (*p* = 0.38) cohesin peaks at a 5-kb resolution. Two-sided *t* test. **e** Chromatin compaction scores around DICs, all cohesin sites and all enhancers (summit ± 500 kb). ΔDLR (E2 vs. control) was calculated at a 25-kb resolution. **f** Heatmap of ChIP-seq reads at DICs (peak summit ± 2.5 kb). CTCF (E2+) signal was used for sorting the order. DICs were divided into HC-DICs (gray bar) and LC-DICs (black bar). E2−, control; E2+, estrogen treated. Source data are provided in the Source Data file. **g** Average binding profiles (summit ± 1.5 kb) of Rad21 and TFs on HC-DICs (upper) and LC-DICs (lower). Reads were normalized relative to the whole genome. The same *y*-axis scale was used for each protein. **h** Read distribution of Rad21 and TFs around *MREG* and *PAK4* loci. Areas shaded in pink and light yellow indicate LC-DICs and HC-DICs, respectively. All reads were normalized relative to the whole genome. **i** Hi-C contact map (5-kb resolution) around *MREG*. Arrows show the disappearance of chromatin interactions after E2 stimulation. The loop anchor is shown as a black bar in 1 h.

MCF-7 cell, when treated with the transcriptional stimulator estradiol^18^, is a widely used model for investigating the transcription-dependent perturbation^6^. We prepared ChIP-seq data of cohesin (Rad21), cohesin loader (MAU2), CTCF and several TFs (ER, CBP, P300, AFF4, TAF1) from MCF-7 cells treated with vehicle (control, or Ctrl) or estradiol (E2, 45 min). The statistics and quality metrics of ChIP-seq and RNA-seq data generated in this study are summarized in Supplementary Data 1–2. All datasets, including our data and public data, are listed in Supplementary Tables 1–2. In total, we obtained 76,668 and 89,111 peaks as cohesin binding sites in the E2 and control data, respectively. Next, we examined the stimulation-dependent cohesin sites (Fig. 1a). Although the total number of cohesin peaks decreased after E2 stimulation, the proportion of peaks that increased (9.3%) after stimulation (log-fold change of peak intensity Mvalue^19^ > 0.5) was larger than the one that decreased (6.2%) (M value < −0.5) (Fig. 1a, bottom). We also found that around 40% (36.3% for E2, 41.2% for control) of cohesin peaks did not overlap with CTCF peaks (Supplementary Fig. 1a). Such ‘cohesin-non-CTCF sites’ (hereafter, CNCs) overlapped with peaks of the enhancer markers P300 and CBP (Supplementary Fig. 1b), which is consistent with an earlier ChIP-seq study^6^. The cohesin loader MAU2 also preferred enhancer sites. In fact, 88.7% of CNCs with enhancer markers overlapped with MAU2, and MAU2 was localized at enhancer sites with and without cohesin binding (Supplementary Fig. 1c, d). This result implies the role of MAU2 in enhancer activity and chromatin interaction, which can precede cohesin localization.

We classified cohesin sites based on gene annotation information (Supplementary Fig. 1e). We defined ‘intragenic cohesin sites’ as sites located within gene bodies, with the exception of transcription start sites (TSSs), transcription end sites (TESs) and alternative promoters. As a result, 13.8% of cohesin sites were identified as intragenic ones, 19.6% of which were overlapped with enhancers annotated by Fantom5^20^. We did not observe a difference in the proportion of up- or down-regulated cohesin peaks between intergenic and intragenic sites (Fig. 1a, lower panel). To investigate the correlation of cohesin binding and transcription activation, we conducted the ChIP-seq of RNA polymerase II (Pol2, unphosphorylated), and RNA pol II CTD serine-2 phosphorylation (Pol2ser2) that represents transcription elongation activity^21^. We identified 499 E2-responsive genes for which Pol2ser2 signal was increased after E2 stimulation (Methods). We then validated these genes by RNA-seq and confirmed that their expressions were mostly up-regulated in response to E2 stimulation (Supplementary Fig. 1f). Based on 499 E2-responsive genes, we identified 4346 intragenic cohesin peaks, 976 (22.4%) of which were decreased after stimulation (Fig. 1b, Supplementary Fig. 1g). The decrease of cohesin binding at DICs was also illustrated in Supplementary Figs 1h–j. Because our main interest is the negative correlation between active transcription and the signal intensity of intragenic cohesin^10^, we focused on the intragenic cohesin sites with decreased peak intensity after E2 stimulation. Hereafter we refer to these sites as DICs. Of the E2-responsive genes, 53.5% (267/499) contained one or more DICs. We found that almost all (97.3% by RefSeq reference) DICs were located in intronic regions (Fig. 1c). While previous studies focused on transcription factor binding on exons^22,23^, our analysis implies a function of DICs at introns whose mechanism remains unrecognized^24^.

Next, we investigated the correlation between decreased cohesin binding and levels of chromatin interaction using Hi-C data (GSE99451). Aggregate peak analysis (APA)^25^ showed that chromatin interactions centered on DICs were weakened by E2 treatment (*p* < 10^−11^, two-side *t* test), whereas no difference (*p* = 0.38) was observed for all cohesin sites (Fig. 1d). These results suggested that at least some intragenic cohesin was required for chromatin loop formation, which was disrupted due to the induction of transcription^10^. In contrast to the positive regulation of gene expression by CNCs^5^, DICs possibly function negatively for gene expression. We then applied DLR (distal-to-local interaction ratio) and ICF (inter-chromosomal fraction of interactions) metrics^10,26^ to represent locus-specific changes in intra- and inter-chromosomal interactions, respectively. The difference (Δ) for DLR (or ICF) between two Hi-C samples represents chromatin compaction (negative value) or de-compaction (positive value). ΔDLR showed a positive value at DICs (Fig. 1e). In contrast, ΔDLR had a negative peak at all cohesin sites, whereas all enhancers showed no enrichment. Chromatin compaction at all cohesin sites could be explained by more frequent *cis*-regulatory interactions after estrogen stimulation. Conversely, DICs did not show a clear difference compared to all cohesin sites for ΔICF (Supplementary Fig. 1k). These results suggested that DICs were involved in intra-chromosome decompaction, creating a more open architecture around DICs.

### Classification of LC-DICs and HC-DICs

We next investigated the binding pattern of cohesin and other TFs, including the estrogen receptor (ER). We found that DICs could be clearly classified into two categories: HC-DICs (high CTCF binding) and LC-DICs (low CTCF binding), in which strong and weak (or no) CTCF peaks co-localized, respectively (Fig. 1f). LC-DICs had a higher probability of co-binding with many TFs as compared with HC-DICs (Fig. 1f, g). This tendency was similar, but not identical, to cohesin peaks in the other regions. For example, cohesin localized with strong CTCF on promoters, where many TFs also bound^5,27^ (Supplementary Fig. 2a). A majority of intergenic cohesin sites (possibly insulator sites or TAD boundaries) did not show enrichment of TFs (Supplementary Fig. 2b). Moreover, the TFs on LC-DICs (Fig. 1g, Supplementary Fig. 2c, except MAU2 and P300), including 16 publicly available TFs (Supplementary Table 3), were increased after E2 treatment. This suggested that enhancer markers MAU2 and P300 were localized to LC-DICs even before stimulation, whereas other TFs (including another enhancer marker CBP) were recruited by E2 stimulation. In addition, we observed increased ER, CBP and CTCF signals on HC-DICs (Fig. 1g), implying the role of CTCF for the estrogen-response transcription there^28,29^. We also divided all cohesin sites into low-CTCF (i.e., CNCs) and high-CTCF ones for comparison with LC-DICs and HC-DICs. Using the APA analysis, we observed the weakened interactions in both LC- and HC-DICs, but not in CNCs or high-CTCF cohesin sites (Supplementary Fig. 2d).

Figure 1h showed examples of two E2-responsive genes (*MREG* and *PAK4*; see Supplementary Fig. 2e for publicly available TFs). For instance, at the *MREG* locus, there were both HC-DICs and LC-DICs, the former co-localizing with strong CTCF signals but almost no TFs, while the latter corresponding to frequent bindings of many TFs yet without strong CTCF signals. Overall, at LC-DICs, the peak intensity of cohesin decreased after E2 stimulation, whereas that of many TFs increased. Consistently, for the E2-activated gene *MREG* (Supplementary Fig. 3a), we could also clearly observe the weakened interactions (Fig. 1i) and the chromatin decompaction (Supplementary Fig. 3b).

More genomic characteristics were detected by motif analysis (Supplementary Figs. 3c, d). Not surprisingly, all types of cohesin showed the motifs of CTCF and CTCFL (BORIS). Specifically, LC-DICs were highly enriched for motifs of the forkhead box (FOX) protein family, which is responsible for remodeling chromatin structure^30^ and controlling transcription^31^. Of note, FOXA1 is a pioneer factor before ER activation in MCF-7 cells^32^. Meanwhile, HC-DICs showed motifs for transcription repressors including the tumor suppressor gene *HIC1*, implying a possible role for HC-DICs in transcription repression. Taken together, these results highlighted the unique features of LC-DICs and HC-DICs relative to other cohesin sites.

### Characterization of LC-DICs as enhancers

**Fig. 2:**
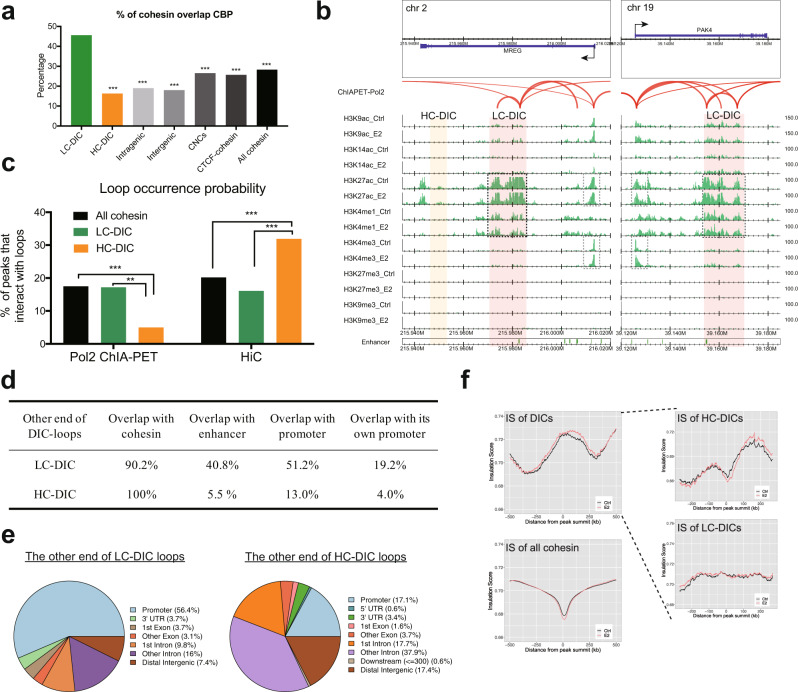
Enhancers and loops on DICs. **a** The proportion of cohesin overlap with CBP peaks. Fisher’s exact test (two-sided) was used between LC-DICs and other cohesin sites. **p < 10^−5^, ***p < 10^−10^. The exact p-values from left to right are 1.5 × 10^−10^, 1.1 × 10^−31^, 1.4 × 10^−37^, 9.9 × 10^−17^, 2.0 × 10^−15^, 9.8 × 10^−14^. Source data are provided in the Source Data file. **b** Genomic binding of multiple histone markers. Enhancer markers H3K27ac and H3K4me1 (black dashed box) were enriched at LC-DICs. Gray dashed boxes indicate histone markers for promoters. Red arcs show Pol2-mediated loops. **c** Loop occurrence probabilities on DICs, calculated based on Pol2 ChIA-PET or Hi-C data. We used Fisher’s exact test (two-sided) to do statistical comparisons. *p < 0.05, **p < 0.01, ***p < 0.001. For ChIA-PET: all cohesin vs. HC-DIC *p* = 5.0 × 10^−5^, LC-DIC vs. HC-DIC *p* = 0.0013; For Hi-C: all cohesin vs. HC-DIC *p* = 0.0003, LC-DIC vs. HC-DIC *p* = 9.0 × 10^−5^. Source data are provided in the Source Data file. **d** The percentage of HiC loops (LC-DICs and HC-DICs) that interacted with cohesin, enhancers, promoters or the promoter of their host gene. **e** Genomic distribution of the other end of DIC-loops as generated by ChIPseeker. Source data are provided in the Source Data file. **f** Insulation scores (IS) for each 25-kb genomic region around DICs or all cohesin sites (summit ± 500 kb). DICs were further divided (dashed line) into HC-DICs and LC-DICs. Source data are provided in the Source Data file.

The binding of the enhancer markers CBP and P300 was frequently observed at LC-DICs (Fig. 1f–h). We confirmed that a significantly higher percentage of LC-DICs overlapped with CBP binding as compared with other cohesin sites (Fig. 2a, Fisher’s exact test). In addition, LC-DICs were also enriched for enhancer markers H3K27ac and H3K4me1 as well as FANTOM5 enhancers^20^ (Fig. 2b, Supplementary Fig. 4a; publicly available data). In contrast, few HC-DICs were annotated as enhancers (16.3% overlap CBP as shown in Fig. 2a, less enrichment of enhancer marker as shown in Fig. 2b and Supplementary Fig. 4a). Moreover, although intergenic cohesin also (including both CTCF-dependent and -independent) in conjunction with many TFs, they were not enriched for enhancer markers (Fig. 2a, Supplementary Fig. 4b). This is consistent with the finding that only 18% of intergenic cohesin co-bound with CBP, which is reasonable because only a subset of intergenic cohesin sites serves as enhancers.

### Characterization of chromatin loops on DICs

To explore DIC-mediated loops, we investigated what kind of chromatin loci interacted with LC-DICs and HC-DICs. Remarkably, when analyzing the Pol2-mediated chromatin loops identified by ChIA-PET (GSE33664), LC-DICs contained multiple Pol2 loops that interacted with the TSS of the host gene (Fig. 2b, red arcs), whereas HC-DICs at the *MREG* locus did not have Pol2 loops (17.2% vs. 5.0% in Fig. 2c). To further investigate this tendency, we also analyzed DIC-anchored loops detected by Hi-C data (GSE99451). Interestingly, this result was in directly opposite between the Hi-C and ChIA-PET Pol2 loops (Fig. 2c, Fisher’s exact test). HC-DICs had a significantly lower occurrence probability with respect to Pol2-mediated chromatin loops, compared to LC-DICs (*p* = 0.0013) or all cohesin sites (*p* < 10^−4^). In contrast, HC-DICs exhibited a significantly higher occurrence with respect to Hi-C loops, as compared with LC-DICs (*p* < 10^−4^) or all cohesin (*p* = 0.0003). We also compared loops with CTCF ChIA-PET data (GSE39495) and found that over 81% of HC-DICs overlapped with CTCF loops (27% for LC-DICs). This result suggested that LC-DICs were anchored by chromatin loops with Pol2 and other TFs, and function as enhancers in a CTCF-independent manner. HC-DICs were more likely to interact with CTCF to form chromatin loops that participate in chromatin architecture independently of the Pol2 machinery.

We then investigated the other anchor sites of the DIC-mediated loops. The other anchor sites of DIC-mediated Hi-C loops also overlapped with cohesin, which also showed a decreasing tendency (Supplementary Fig. 5a). As shown in Fig. 2d, e, LC-DIC loops (ChIA-PET and Hi-C) mainly interacted with enhancers (40.8%) or promoters (51.2%), which was confirmed by high enrichment of active histone markers (Supplementary Fig. 5b). We also observed that only a subset of LC-DIC loops (19.2%) interacted with the promoter of their host genes, suggesting that LC-DICs also contribute to the regulation of distant non-host genes, possibly as intragenic enhancer sites. In contrast, most of the HC-DIC loops did not interact with promoter or enhancer sites (Fig. 2d, e). Instead, over half of these sites interacted with intronic regions (Fig. 2e; example loci are shown in Supplementary Fig. 5c). In summary, these results suggested that LC-DICs participated in transcriptional regulation, whereas HC-DICs were more likely to connect the intronic regions of two genes.

We also examined the insulation score (IS) from Hi-C data, for which a lower value indicates more insulated regions, e.g., TAD boundaries. Although the IS profile showed a clear valley at all, intergenic and intragenic cohesin sites (Fig. 2f, Supplementary Fig. 5d), it peaked at DICs (Fig. 2f, top left). Interestingly, the IS profile for HC-DICs showed bimodal peaks around a small valley, whereas there was neither a peak nor valley at LC-DICs (Fig. 2f, lower right). These results suggested that LC-DICs possibly act as enhancers within TADs and that HC-DICs participate in the formation of boundaries, which is consistent with our loop analysis described above.

### Assessment of Pol2 stalling on DICs

**Fig. 3:**
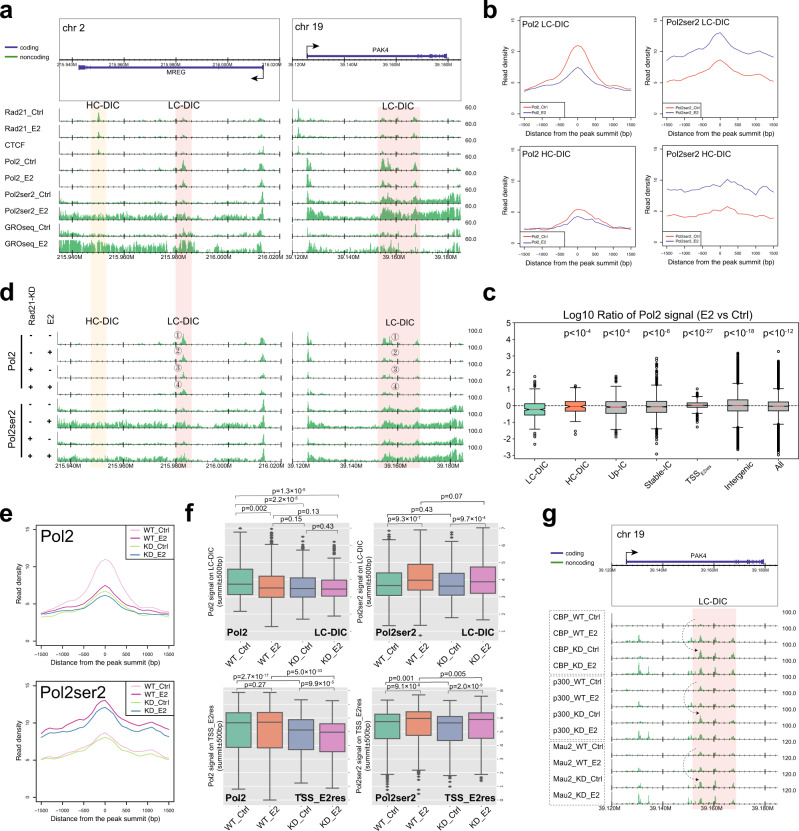
Pol2 pauses on LC-DICs. **a** Genomic binding of Pol2 and Pol2ser2 and GRO-seq data for *MREG* and *PAK4* loci. **b** Binding profiles of Pol2 and Pol2ser2 on LC-DICs or HC-DICs, under control and E2 conditions. Reads were normalized relative to the whole genome. **c** The log_10_ value of the ratio E2/Ctrl with respect to the Pol2 signal on various cohesin sites. *p* values (Mann–Whitney *U* test, two-sided) were calculated between LC-DICs (*n* = 417) and other cohesin sites including HC-DIC (*n* = 141, *p* = 5.0 × 10^−5^); Up-IC (up-regulated intragenic cohesin, *n* = 680, *p* = 7.6 × 10^−5^); stable-IC (unchanged intragenic cohesin, *n* = 2800, *p* = 4.7 × 10^−9^); TSS_E2res_, (TSS of E2-responsive gene, *n* = 1422, *p* = 2.4 × 10^−28^); intergenic cohesin (*n* = 34,566, *p* = 1.7 × 10^−19^) and all cohesin sites (*n* = 96,218, *p* = 1.6 × 10^−13^), where n is the number of cohesin sites. Box plots indicate the interquartile range IQR (25–75%) with a line at the median. Whiskers indicate 1.5 times the IQR. Black circles represent outliers. **d** Visualization of Pol2 and Pol2ser2 ChIP-seq data around *MREG* and *PAK4*. MCF-7 cells were treated as indicated. WT, wild type; KD, Rad21 knockdown; Ctrl, control; E2, estrogen. **e** Pol2 and Pol2ser2 binding profiles around LC-DICs (summit ± 1.5 kb) under four different conditions. **f** Quantitative comparison (Mann–Whitney *U* test, two-sided) of Pol2 and Pol2ser2 signals on LC-DICs (*n* = 417) or TSSs of E2-responsive genes (TSS_E2res, *n* = 1422) under four different conditions. Multiple testing correlation with Benjamini-Hochberg method was used. Box plots indicate the interquartile range IQR (25–75%) with a line at the median. Whiskers indicate 1.5 times the IQR. The black diamond symbols represent outliers. Source data are provided in the Source Data file. **g** ChIP-seq results for CBP, P300 and MAU2 on *PAK4* gene in WT and Rad21 knockdown MCF-7 cells with the absence and presence of E2 stimulation. Dashed arrows show increased binding events in cohesin-deficient cells.

Pol2 is released from promoter-proximal pausing to transcribe the entire gene body, although it may be temporarily paused by roadblocks within gene bodies^12^. To test whether DICs can function as roadblocks, we investigated the Pol2 enrichment at DICs using our Pol2 (unphosphorylated CTD) and Pol2ser2 (ser2 phosphorylated CTD) ChIP-seq data (Supplementary Fig. 6a, b). Our Pol2ser2 and public global nuclear run-on sequencing data (GRO-seq, GSE99508) showed that transcription elongation was activated by E2 (Fig. 3a). Moreover, we found that Pol2 peaked at LC-DICs, and its intensity decreased after E2 stimulation (Fig. 3a, b), possibly due to the release of paused Pol2. This tendency towards a decrease in Pol2 binding was statistically significant as compared with the other cohesin sites (Fig. 3c, Supplementary Figs. 6c–e). Public Pol2 ChIP-seq datasets further illustrated the decreased Pol2 (Supplementary Fig. 6f). Given that the binding of most TFs was increased by E2 stimulation at LC-DICs (Fig. 1g, h), cohesin binding that decreased at LC-DICs was more likely to be accordant with Pol2, rather than TF binding. In contrast, Pol2ser2 was increased on all DICs due to transcription activation (Fig. 3b, right panel). Pol2ser2 also exhibited peak-like enrichment at LC-DICs, which was increased by E2 stimulation (Fig. 3a, b). This is remarkable given that Pol2 enrichment at LC-DICs decreased significantly after E2 stimulation (Fig. 3b, c). In addition, whereas Pol2 binding on TSSs of DIC-host genes did not show any difference after E2 stimulation, the intensity of Pol2ser2 on TSSs increased (Supplementary Fig. 6g). These results were consistent with our hypothesis that Pol2 temporarily stalls within DICs, which function as roadblocks, and then is released by the loss of cohesin.

### Knockdown analysis of cohesin revealed that cohesin had a function at DICs

Although we observed cohesin binding at DICs that was synchronized with Pol2 binding and was negatively correlated with gene expression, there is still a possibility that cohesin is “passively” localized to DICs and therefore does not have any active role in gene expression. To determine whether cohesin functions in the pausing of Pol2 at LC-DICs, we prepared Pol2 and Pol2ser2 ChIP-seq data in the absence (WT, wildtype) and presence (KD, knockdown) of Rad21-specific siRNA (siRad21) to generate Rad21 knockdown (Supplementary Fig. 7a, b) and investigate the effect at DICs. Pol2 binding before E2 stimulation (Ctrl, control for estrogen treatment) was significantly decreased by siRad21 (example region: site 3 vs. 1 in Fig. 3d; all LC-DICs: KD_Ctrl as compared with WT_Ctrl in Fig. 3e, f) and had a similarly low level of binding in E2-treated (E2, estrogen-stimulated) wild-type cells (example region: site 2 in Fig. 3d, all LC-DICs: WT_E2 in Fig. 3e, f). Supplementary Fig. 7c–e also illustrated the decrease of Pol2, as evidenced by both replicates and the knockdown of cohesin loader NIPBL. Importantly, the Pol2 tendency at LC-DICs is distinct from the one at TSS of E2-response genes (TSS_E2_res_). For example, the cohesin KD under E2 (example region: Fig. 3d; all LC-DICs: KD_E2 vs WT_E2 in Fig. 3e, f) showed unchanged Pol2 at LC-DICs (*p* = 0.13), but significant changes at TSS_E2_res_ (*p* < 10^−32^). These results suggested that cohesin binding on LC-DICs is not passive and plays a role related to the Pol2 binding level. Pol2 binding in KD_Ctrl cells was not affected by E2-stimulation (example region: site 4 vs. 3 in Fig. 3d; all LC-DICs: KD_E2 as compared with KD_Ctrl in Fig. 3e, f), possibly because Pol2 that was paused in WT_Ctrl cells had already been released in KD_Ctrl cells. Importantly, the effect of siRad21 on the Pol2 signal at TSSs of E2-responsive genes was distinct from LC-DICs, in which Pol2 binding did not change significantly after E2 stimulation in WT cells (from WT_Ctrl to WT_E2 in Fig. 3d, f) but decreased after siRad21 in stimulated cells (from WT_E2 to KD_E2 in Fig. 3d, f). These results also supported the model that on LC-DICs the loss of cohesin binding causes the release of paused Pol2. On HC-DICs, we did not observe changes with comparable significance (Supplementary Fig. 7f).

Interestingly, siRad21 did not largely affect Pol2ser2 binding. Pol2ser2 levels on LC-DICs were not obviously different between WT and siRad21 cells (Fig. 3d–f, Supplementary Fig. 7g, h). In KD_Ctrl cells, there was no more stalling at LC-DICs, but there was also no stimulating effect of E2; thus Pol2ser2 did not change from WT_Ctrl to KD_Ctrl. In KD_E2 cells, transcription was activated by E2 stimulation but was limited by the loss of cohesin on TSSs, and thus Pol2ser2 binding changed slightly from WT_E2 to KD_E2. To explore changes in the expression level of genes that harbor LC-DICs after siRad21 treatment, we conducted RNA-seq with siRad21 (Supplementary Fig. 7i). Without E2 treatment, siRad21 did not significantly affect gene expression (*p* = 0.23, KD_Ctrl as compared with WT_Ctrl). After E2 treatment, siRad21 moderately affected gene expression (*p* = 0.0057, KD_E2 as compared with WT_E2). Indeed, only a small subset (~10%) of LC-DIC-host genes (Fisher’s exact test *p* > 0.1 compared with other E2-response genes) were identified as differentially expressed genes. It is possible because only a subset of Pol2 that had paused on LC-DICs represented productive Pol2. We also quantitatively compared Pol2 and Pol2ser2 signals under four different conditions on various cohesin sites (Fig. 3f, Supplementary Fig. 7j). The results confirmed the significantly reduced binding of Pol2 in WT_E2, KD_Ctrl and KD_E2 as compared with WT_Ctrl cells (Fig. 3f, Mann–Whitney *U* test, one-sided). Such a tendency was distinct from those involving the TSSs of E2-responsive genes, up-regulated and non-changed intragenic cohesin, other cohesin sites, and also other enhancer sites. Our results suggested the role of cohesin at LC-DICs which is different from the known roles of cohesin sites.

In Fig. 1g, h, we showed the elevated binding of many TFs on DICs. To investigate whether the increased binding of multiple TFs is caused by a decrease in cohesin binding, we generated ChIP-seq data for CBP, P300 and MAU2 from siRad21 cells. Remarkably, a cohesin deficiency resulted in stronger binding of those TFs at LC-DICs, which surpassed the level in ER-stimulated WT cells (dashed arrow in Fig. 3g, Supplementary Fig. 8a, b). In contrast, there was the little effect at the other intragenic enhancer site (Fig. 3g). Considering that E2 stimulation recruits TFs by estrogen responsive elements in WT cells, the increased binding of TFs in non-E2-stimulated siRad21 cells suggested that cohesin suppresses TF binding at LC-DICs in some way, and this suppression is removed by the loss of cohesin. In combination with the chromatin de-compaction by E2 stimulation shown in Fig. 1e, the increased binding of TFs at LC-DICs can be explained, at least in part, by a more accessible chromatin structure near the LC-DICs, which is caused by the disruption of cohesin-mediated interactions.

### Machine learning analysis of DIC features

Although we manually defined the criteria for DICs in the analysis above, we also wondered whether DICs can be automatically isolated based on various genomic features obtained from our multi-omics information. To this end, we implemented machine learning (ML) (Supplementary Fig. 9a), which provides a more objective approach to study DICs. We generated an integrated data matrix consisting of 175 features from genomic, transcriptomic and epigenomic data for all cohesin sites (Supplementary Table 4; Methods). Especially, this matrix includes features related to genomic location (e.g., intragenic or TSS) and perturbation by E2 stimulation such as M value and ΔDLR. Supplementary Fig. 9b showed a Pearson correlation heatmap followed by hierarchical clustering between all-by-all features for DICs or all cohesin sites. The 175 features resulted in clear clusters both among DICs and among all cohesin sites (dashed boxes of different colors). We annotated the clusters as promoter, enhancer, enhancer-promoter interaction (E-P), insulator, and chromatin architecture. As compared with all cohesin sites, DICs showed lower co-binding tendency in the promoter cluster and higher co-binding in the enhancer and E-P clusters. This showed the effectiveness of our matrix in distinguishing DICs from other cohesin sites.

**Fig. 4:**
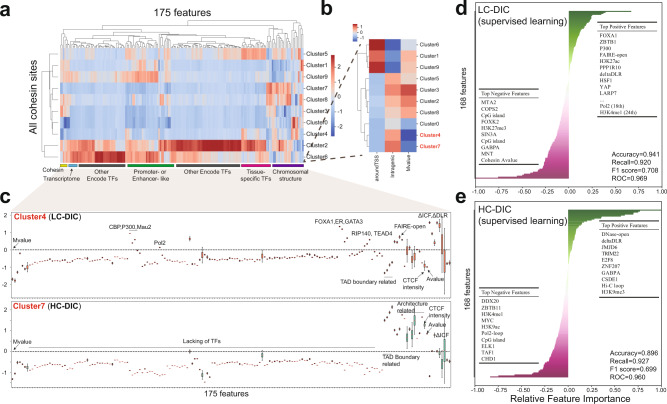
Machine learning methods to classify DICs. **a** Cluster heatmap by k-means (k = 10) methods. The 175 features were roughly classified into different groups. Source data are provided in the Source Data file. **b** Magnified view of the heatmap area shown in A indicates three important features for these 10 clusters: “aroundTSS” indicates whether a cohesin site was near TSS; “intragenic” indicates whether a cohesin site was within a gene body; “M value” is the log_2_ ratio of Rad21 peak density between the E2 and control group. **c** The boxplot of each feature for cluster 4 (*n* = 11,920) and 7 (*n* = 33,158), where n is the number of cohesin sites. The *y* axis shows the normalized (*z* score) values of binary and continuous features. Box plots indicate the interquartile range IQR (25–75%) with a line at the mean. Whiskers indicate 95% confidence intervals. **d**, **e** The importance of individual features from the logistic regression model for (**d**) LC-DICs and (**e**) HC-DICs. Top positive and negative features are listed inside the plots. Model performance is indicated in the bottom right.

Similar to the previous study of CNCs^5^, we applied unsupervised k-means clustering (k = 10) to the matrix and obtained 10 clusters for all cohesin sites (cluster 0−9, Fig. 4a, Supplementary Fig. 12), among which only cluster 4 and cluster 7 showed intragenic cohesin binding that decreased after E2 stimulation, indicating the DIC-like clusters (Fig. 4b). We identified the following characteristics of cluster 4 (Fig. 4c, upper): (1) co-binding with tissue-specific TFs (e.g., ER and FOXA1), (2) enrichment of enhancer markers and Pol2, (3) relatively low intensity of cohesin and CTCF peaks and (4) chromatin de-compaction. Therefore, cluster 4 represented the LC-DIC-like cluster. In contrast, cluster 7 (Fig. 4c, lower) showed the following characteristics: (1) lack of TF co-binding, (2) high intensity of CTCF peaks and (3) highly related to topological boundaries and chromatin architecture features (e.g., TAD borders, Hi-C loops). Therefore, cluster 7 represented the HC-DIC-like cluster. Compared with “CNC-like” intragenic cohesin sites^5,6^ (clusters 2, 3 and 8; Supplementary Fig. 12), cluster 4 (LC-DICs) co-localized only with enhancer markers and several master regulators (FOXA1, ER and GATA3), and therefore it is distinct from typical *cis*-regulatory modules (CRMs) at which many TFs co-localize. In contrast, cluster 7 (HC-DICs) consists of a cluster of intragenic cohesin sites that tend to be localized to open chromatin, are highly de-compacted and contain loops but are strongly negatively correlated with TFs. Therefore, they may be associated with a more universal chromatin structure that is required for proper gene transcription.

To further explore the importance of genomic features related to DICs, we applied modeling of supervised ML (logistic regression, support vector machine, and random forest) to predict LC- and HC-DICs from all cohesin sites in a binary manner (labeled by 0 or 1). In this analysis, the input matrix consisted of 168 features (features related to genomic location, cohesin changes and CTCF signal were excluded). We selected chromosomes 16 to 22 for testing, and the remaining chromosomes were divided into training and validation by five-fold cross-validation (see Methods). Because DICs are a small subset of all cohesin sites, we used SMOTE over-sampling^33^ to deal with such “imbalanced” classifications. The trained model that was based on logistic regression achieved the best performance overall as compared with the others (Supplementary Figs. 9c, d) and also performed adequately with the test data (Supplementary Fig. 9e). Finally, we identified important features for the prediction of LC-DICs and HC-DICs by calculating the relative feature importance from the trained model (Fig. 4d, e). LC-DICs were positively associated with (1) enhancer markers (H3K27ac, H3K4me1, P300); (2) Pol2 peaks, the Pol2-pausing regulator (LARP7)^34^ and a transcriptional repressor (ZBTB1);^35^ (3) tissue-specific regulators (FOXA1^32^, HSF1^36^, ER^18^). Both LC-DICs and HC-DICs were positively associated with open chromatin (FAIRE-open, DNase-open) and chromatin de-compaction (ΔDLR), and were negatively associated with H3K27me3 and CpG island levels. HC-DICs, in particular, showed positive features of Hi-C loops but negative features of Pol2 loops and TF binding, which is consistent with our analysis above, indicative of the TF-independent chromatin de-compaction. Taken together, the application of machine learning successfully isolated a special subset of cohesin sites corresponding to DICs, which also provided additional characteristics for DICs.

### Characterization of DIC tissue specificity

**Fig. 5:**
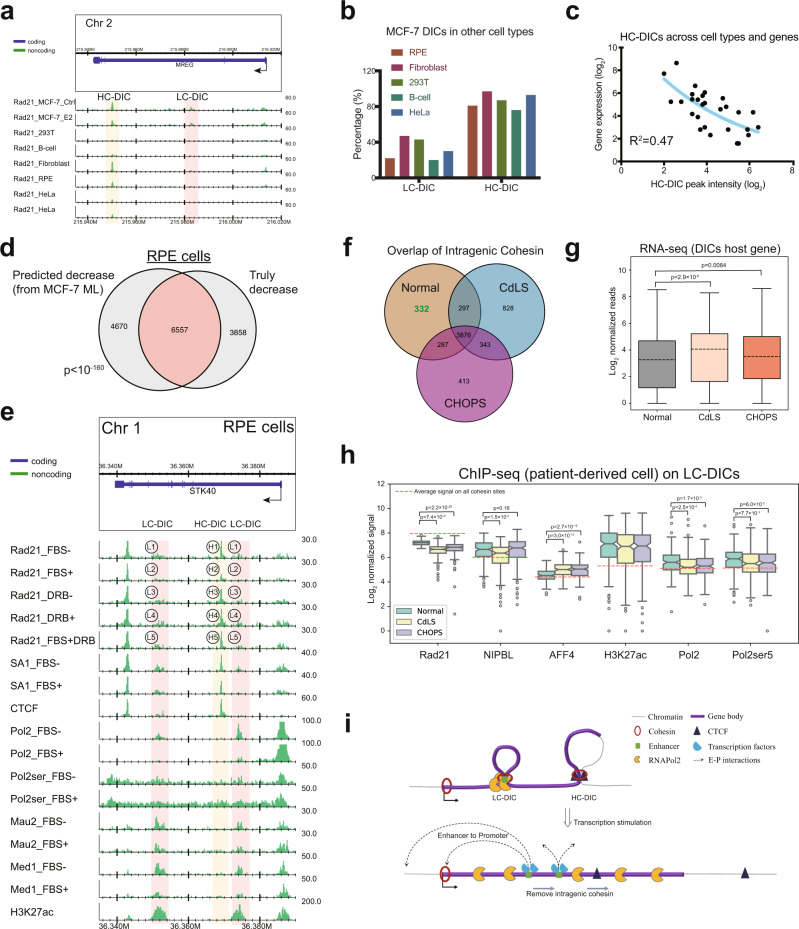
DICs in other cell types. **a** Rad21 ChIP-seq of the *MREG* locus in various cell lines (MCF-7, 293 T, B-cells, Fibroblasts, RPE, HeLa). HC-DICs were observed in other cells but not LC-DICs. **b** The percentage of MCF-7 DICs that could be found in other cell types. Source data are provided in the Source Data file. **c** Relationships between peak density of HC-DICs and the expression of their host genes. Gene expression data for the different cell types were obtained from the GTEx database. **d** Overlap between DICs that were predicted relative to those that were experimentally isolated; *p*-values were calculated with the hypergeometric test (two-sided, *p* = 2.4 × 10^−161^). **e** ChIP-seq data of the example locus in RPE cells. L1-5 indicated LC-DIC sites and H1-5 indicated HCDIC sites. **f** Overlap of intragenic cohesin sites in normal and CdLS- and CHOPs-derived fibroblast cells. **g** Expression of genes associated with DICs (paired *t* test, one-sided, *n* = 332) in Normal, CdLS (*p* = 2.9 × 10^−6^) and CHOPs (*p* = 0.0084) cells. Box plots indicate the interquartile range IQR (25–75%) with a line at the median. Whiskers indicate 1.5 times the IQR. **h** Binding of Rad21, NIPBL, AFF4, H3K27ac, Pol2 and Pol2ser5 on LC-DICs. Box plots indicate the interquartile range IQR (25–75%) with a line at the median. Whiskers indicate 1.5 times the IQR. Dashed lines represent the average binding signal of each TF on all cohesin sites. Mann–Whitney *U* test (*n* = 332, two-sided) was used to compute *p* values. Black circles represent outliers. Source data are provided in the Source Data file. **i** Proposed model for our DICs. Briefly, intragenic loops formed by LC-DICs pause Pol2, whereas HC-DICs form CTCF-mediated loops. Transcription stimulation could displace DICs and disrupt DIC loops, consequently releasing the Pol2 pause. The exposed enhancer sites on LC-DICs then bind their respective TFs and interact with promoters.

As DICs were enriched by many tissue-specific factors, we wondered whether our observations about DICs were consistent with other tissues or cell types. We generated Rad21 ChIP-seq for 293 T cells (kidney), B-cells (lymphocytes), human skin fibroblast cells, RPE (retinal pigmented epithelium) cells, and HeLa cells (cervical cancer). Cohesin peaks at MCF-7 derived LC-DICs were more specific in MCF-7 cells, whereas cohesin peaks at HC-DICs were more ubiquitous across cell types (Fig. 5a, b). Thus, LC-DICs are likely to play a role in tissue-specific transcription. On the other hand, considering the intragenic CTCF also regulates transcription^23,37^, we then asked whether the ubiquitous HC-DICs, which have high-level CTCF, can affect transcription across cell types. As a result, the peak intensities for Rad21 at HC-DICs were negatively correlated with transcription levels of their host genes (Fig. 5c, Supplementary Fig. 10a), suggesting that genes with stronger HC-DIC binding had lower transcription activities. Therefore, HC-DICs may also participate in transcription regulation, which is consistent with our motif analysis in Supplementary Fig. 3c.

To confirm whether DICs in other cell types also exhibit similar characteristics, we performed ChIP-seq experiments on RPE cells with FBS (fetal bovine serum) and DRB (5,6-dichloro-1-β-d- ribofuranosylbenzimidazole), which function as a stimulator and inhibitor of transcription^38^, respectively. First, we tested if the ML model trained by MCF-7 data was applicable to the RPE data. We used 25 features that were available for both MCF-7 and RPE cells to predict whether the binding of intragenic cohesin was decreased or not after transcription stimulation (Fig. 5d). The predicted DICs overlapped extensively with the experimentally determined ones (*p* < 10^−160^, hypergeometric test), indicating that DICs exhibited some common rules across cell types. Then we identified DICs of stimulation-responsive genes in RPE cells (Supplementary Fig. 10b), and the decreased Rad21 was confirmed by replicates as shown in Supplementary Figs. 10c–e. Similar to DICs in MCF-7 cells, RPE-derived DICs also showed tissue-specific binding patterns (Supplementary Fig. 10f). FBS stimulation decreased the intensity of cohesin (Rad21 and SA1) at DICs (example region: H1 to H2, L1 to L2 in Fig. 5e; all DICs: Supplementary Figs. 10g), whereas transcriptional inhibition by DRB increased it. In addition, further treatment with DRB (i.e., FBS + DRB) reverted the decrease in cohesin binding caused by FBS stimulation (example region: H2 to H5, L2 to L5 in Fig. 5e; all DICs: Supplementary Fig. 10g). Moreover, RNA Pol2 stalling and the release of paused Pol2 were also observed at LC-DICs in RPE cells (example region: Fig. 5e; all DICs: Supplementary Fig. 10h). In addition, LC-DICs, but not HC-DICs, co-bound with enhancer marks and several TFs. Thus, DICs are not a phenomenon associated only with breast cancer cells, but are found in non-cancer derived human cell lines as well.

### Analysis of DICs in CdLS and CHOPS cells

Finally, we attempted to examine the participation of DICs in the observed phenotypes in individuals with CdLS and CHOPS. To this end, we generated ChIP-seq data for fibroblast cells derived from patients and non-patients (as control)^17^. We overlapped the binding sites of intragenic cohesin in different cell types (Fig. 5f). Whereas most sites were shared among samples, 332 Rad21 sites were absent in both CdLS and CHOPS cells, which we defined as DICs. RNA-seq analysis showed a significant increase in the transcription of DICs (both LC- and HC-DICs) host genes in CdLS and CHOPS cells (Fig. 5g, Supplementary Fig. 11a; paired *t* test *p* < 10^−5^ in CdLS and *p* = 0.0084 in CHOPS), suggesting that the decreased cohesin binding at DICs was correlated with upregulated gene expression in both CdLS and CHOPS. We classified DICs into 185 LC-DICs and 147 HC-DICs based on CTCF signal (from the ENCODE project: id ENCFF757GIM), and we assessed the binding of TFs (Fig. 5h, Supplementary Fig. 11b). Interestingly, at LC-DICs, the peak intensity of AFF4 (causative gene of CHOPS) increased in both CdLS and CHOPS cells, whereas lower binding of NIPBL (causative gene of CdLS) was observed only in CdLS cells (Fig. 5h). Enhancer marker H3K27ac was highly enriched but unchanged among the three cell types, whereas Pol2 and Pol2ser5 (RNA pol II CTD phospho Ser5, which represents paused Pol2) were decreased in both CdLS and CHOPS cells, consistent with our observations in MCF-7 and RPE cells. Taken together, this result suggests that DICs, especially LC-DICs, are involved in abnormal transcription associated with both CdLS and CHOPS. As both CdLS and CHOPs are involved in abnormal Pol2 regulation^39^, LC-DICs might offer a common pathogenetic mechanism. Based on the observations, we concluded that intragenic cohesin sites can be a good candidate to investigate and link the phenotypes of these two cohesinopathy disorders.

## Discussion

Cohesin is thought to be responsible for transcriptional regulation and chromatin folding. Several models have been proposed to explain its functions. Cohesin can mediate enhancer-promoter loops with the mediator complex or function as a blocker between enhancer and promoter in conjunction with insulator factor CTCF^40^. Cohesin also participates in the formation of chromatin topological structures via the loop extrusion model^8^. A recent paper reported that transcription stimuli such as IFN-beta in THP-1 cells can displace cohesin from chromatin^10^, which attracted our interest. Here, we focused on intragenic cohesin, a subset of cohesin that has not been discussed by previous research. Of note, we emphasized the negative regulation of gene expression by cohesin-mediated chromatin loops, whereas most of the previous studies implicitly assumed the positive regulation. DICs were negatively associated with activated transcription and chromatin compaction. LC-DICs were highly enriched with enhancer markers and paused Pol2, whereas HC-DICs were more involved in the features of chromatin architectures. Importantly, DICs could be found in multiple cell types, especially in CdLS and CHOPS cells, which partly supported the similarities between CdLS and CHOPS.

Chromatin interactions are required not only to facilitate transcription but also for Pol2 pausing^41^. By using siRad21 cells, we observed that the release of Pol2 was related to the loss of cohesin at LC-DICs, which supported our model that intragenic loops formed by cohesin paused Pol2 and that transcription elongation from TSSs could remove such cohesin and then release the paused Pol2 (Fig. 5i). Velasco et al.^23^ has suggested that CTCF-mediated intragenic loops regulate alternative splicing. Other studies^42,43^ have also found that the slowing down of Pol2 elongation is a mechanism of splicing regulation. In our study, we can observe the stalling of Pol2 on LC-DICs, but we did not observe significant changes in the expression of genes that host LC-DICs by siRad21 (Supplementary Fig. 7i). As most LC-DICs were in intronic regions, Pol2 released from LC-DICs might be involved in accurate RNA splicing, which inspires the future study about DICs. Notably, a recent study suggested that intragenic enhancers, in addition to activating genes, also attenuate the transcription of their host genes during productive elongation^12^, which evokes the functional link between LC-DICs and Pol2 pausing. In contrast, HC-DICs showed a high preference for loop occurrence mediated by CTCF, possibly to play a role in topological boundaries (e.g., sub-TADs). Across different cell types and genes, the Rad21 signal at HC-DICs was negatively correlated with the expression of host genes, indicating the role of HC-DICs in restraining transcription. Whereas we observed that more than half of HC-DIC−mediated loops anchored intronic regions of two genes, it was difficult to infer the biological meaning of this because HC-DICs scarcely overlapped with any other TFs. Further biological approaches such as genome editing of HC-DICs of activated genes could be promising in the future.

Modifications at intragenic regions affect transcription events. For instance, intragenic methylation can prevent spurious transcription initiation^11^; Intragenic microRNAs affect the expression of their host genes^13^. Here we present a specific study focusing on intragenic cohesin sites. We also used penalty linear regression followed by univariate linear regression to better understand the changes of cohesin binding in intragenic regions (see Method and Supplementary Fig. 9f–h). Apart from the decreased ones, the increased intragenic cohesin sites seemed to be also correlated with many important features, as they are positively predicted by ER and several TFs. Although we characterized intragenic cohesin sites that showed decreased binding in this study, all types of intragenic cohesin might have a role in transcriptional regulation. In addition, Kowalczyk et al. point out that intragenic enhancers can act as alternative promoters^44^. Our DICs did not overlap with any known alternative promoters. Even though the detailed molecular mechanism is not clear, our results strongly suggest a previously undescribed function of cohesin in intragenic regions with respect to gene expression regulation.

In summary, large-scale multi-omics enabled us to identify a cluster of cohesin DICs in MCF-7 and other cell types. Some tissue-specific DICs (LC-DICs) were related to enhancers and the accumulated Pol2, whereas others (HC-DICs) contributed to chromatin architecture and might attenuate transcription. Our integrated analysis and machine learning approaches indicated distinct characteristics that distinguish DICs from other cohesin binding sites. Based on these genomic, epigenomic and transcriptomic characteristics, we can infer that DICs have distinct functions as compared with other cohesin sites.

## Methods

### Cell culture and treatment

RPE cells^45^, MCF-7 cells (JCRB Cell Bank) and immortalized fibroblast cells (generated in our previous study^17^) were cultured in DMEM containing 10% FBS and 1% penicillin/streptomycin. Before subsequent treatments, RPE cells were cultured in serum-free medium for 48 h and then were incubated in DMEM containing 10% FBS for 30 min. MCF-7 cells were maintained in phenol red−free medium containing charcoal-dextran−stripped FBS (Life Technologies) at 70−80% confluency for 2 days before treatment with 50 nM E2 (beta-estradiol, SIGMA, E2758) for the indicated times. Rad21 stealth siRNAs UUCCACUCUACCUGAUUCAAGCUG (Thermo Fisher Scientific, also used in previous report^4^) were transfected using Lipofectamine RNAiMax (Thermo Fisher Scientific, 13778150) according to the manufacturer’s instructions at 40 h before treatment with E2. DRB (TCI, D4292) was added at 1.5 h before treatment with E2. The effect of cohesin (Rad21)-deficiency was verified by western blot as shown in Supplementary Fig. 7a.

### ChIP and antibodies

Cells were fixed in medium or phosphate buffered saline with 1% formaldehyde at room temperature for 10 min. ChIP experiments were performed as described^46^. ChIP-seq libraries were prepared using NEBNext ChIP-seq Library Prep Master Mix Set for Illumina (New England BioLabs, E6240). Rabbit polyclonal antibody for Rad21 (1:1000 dilution for western blot; 2.5 ug/million cells for ChIP-seq) was obtained from Eurofins Genomics and has been described in^47^. Antibodies for MAU2 (ab46906, 2.5 ug/million cells as dilution) and SA1 (ab4457, 2.5 ug/million cells) were from Abcam. Antibodies for TAF1 (A303-505A, 2.5 ug/million cells) and AFF4 (A302-538A, 2.5 ug/million cells) were from Bethyl Laboratory. CTCF (07-729, 2.5 ug/million cells) antibody was from Merck Millipore. Antibodies (2.5 ug/million cells) for unphosphorylated Pol2 (CMA601), Pol2ser2 (CMA602) and H3K27ac (CMA309) were kindly provided by Dr. H Kimura (TITech), which were used in previous studies^17,48^. Antibody for CBP (606402, 2.5 ug/million cells) was from BioLegend. Antibodies for P300 (sc-585, 2.5 ug/million cells) and Med1 (sc-5334, 2.5 ug/million cells) were from Santa Cruz Biotechnology.

### ChIP-seq analysis

After quality check by FastQC and SSP^49^, ChIP-seq reads were aligned to the human reference genome (hg38) using Bowtie^50^ version 1.2.2 with “-n2 -m1” parameters, by which we considered only uniquely mapped reads and allowed two mismatches in the first 28 bases per read. Peak calling was performed using MACS2^51^ version 2.2.6 with default settings. We used DROMPA^52^ version 3.7.2 to conduct statistical analysis and visualization. For visualization of ChIP-seq binding to particular chromatin regions, reads were normalized relative to total read number, and gene annotation was obtained from NCBI reference sequences (RefSeq; hg38). Read profiles around the sites of interest were plotted with the PROFILE mode of DROMPA, whereas the heatmap of target sites (2.5 kb around the peak summit) was plotted using HEATMAP mode. Genomic distribution in Fig. 2e was plotted by ChIPseeker^53^. Downstream analysis, such as peak overlap, was performed by Bedtools^54^ version 2.29.2 and Samtools^55^ version 1.9. Sources for all ChIP-seq data and other next-generation sequencing data (including our data and public data) are listed in Supplementary Tables 1–2.

### Hi-C analysis

All in-situ Hi-C data (control or E2-treated MCF-7 cells with two replicates) were aligned to the hg38 human reference genome. Further analysis was carried out mainly by Juicer^25^ version 1.11.04. All contact matrices were normalized by the KR method in Juicer. Chromatin loops were annotated using the HiCCUPS algorithm with default parameters^25^. The loop regions we used were merged from the results of 5-kb,10-kb and 25-kb resolutions. Aggregate peak analysis (APA) was performed using the ‘apa’ mode of Juicer (5-kb resolution), to measure the enrichment of the Hi-C signal around a set of peaks. The visualization of the contact matrix on the *MREG* locus was accomplished by Matplolib. After correction and normalization, comparable contact matrices were plotted at a 5-kb resolution. We merged two adjacent bins for smoothing. Other Hi-C analyses were performed using HOMER^10^. We made the Tag directory with the “GATC” restriction site sequence. Chromatin compaction scores ΔDLR and ΔICF were calculated for each 5-kb region across the genome (-res 5000) from a 15-kb window size (-window 15,000). Other metrics including PC1, insulation score and TAD boundaries were obtained using HOMER with default parameters. We used the WashU epigenome browser^56^ to visualize Supplementary Fig. 3b.

### ChIA-PET analysis

RNA polymerase II−bound chromatin interactions in MCF-7 cells were extracted from ChIA-PET data (GSE33664). All fastq files were applied to the published pipeline Mango^57^ with default parameters, based on the hg38 reference genome. ChIA-PET interactions were visualized by DROMPA with the parameter ‘-inter’.

### RNA-seq and GRO-seq analysis

Using HISAT2^58^ version 2.2.0, we aligned paired-end RNA-seq reads to the index established from the hg38 reference genome. The output SAM files were converted to BAM files by Samtools. Htseq^59^ version 0.11.3 was then used with default parameters to generate a count table, which describes the number of reads on each gene. We used a GTF file (GRCh38.p12) from GENCODE for gene annotation. Subsequent differential expression analysis was achieved using DESeq2^60^, with its internal normalization. For GRO-seq, alignment was carried out using Bowtie with “-n2 -m1” parameters. The output was preprocessed and visualized using DROMPA.

### Data collection and machine learning

All datasets used in machine learning are listed in Supplementary Table 4. Apart from our data, public omics data in wild-type MCF-7 cells were downloaded mainly from the GEO database, ENA database, ENCODE project, FANTOM5 project, UCSC genome browser and GWAS Catalog database. These data were then overlapped with all 184,140 cohesin peaks. As a result, we obtained 15 continuous features and 160 binary features, the latter of which indicated whether a kind of data was co-localized (1) or not co-localized (0) at a cohesin site. After normalization of continuous features, the big matrix, which consisted of 184,140 rows (cohesin sites) and 175 features, was imported into for different analyses. The features correlation heatmap for all cohesin sites and DICs was made with the R package *corrplot*. We used scikit-learn version 0.22.1 to perform machine learning. Overall, the parameters used in scikit-learn were optimized by grid search with 5-fold cross-validation. For unsupervised learning (k-means), all 175 features were used to fit models. For supervised learning (logistic regression, support vector machine, random forest), we omitted Mvalue, cohesin location and CTCF signal information and then used the remaining 168 features as independent variables ${X}_{i}=({X}_{i1},{X}_{i2},\ldots ,{X}_{{ij}})$, for $i=1,2,\ldots ,184140$ and $j=1,2,\ldots ,168$. Based on whether they were DICs or not, we labeled each cohesin site as 1 or 0 and then used it as a dependent variable ${Y}_{i}\in \{0,1\}$. The conditional probability of logistic regression was calculated as follows:

1 $$P\left({Y}_{i}=1|X={X}_{i}\right)=\;\frac{1}{1+e^{-\left({\beta }_{0}+\mathop{\sum }\nolimits_{j=1}^{168}{\beta }_{j}{X}_{{ij}}\right)}}$$

where ${{{{{{\rm{\beta }}}}}}}_{j}$ is the regression coefficient of each feature. We used training data to do model fitting and used test data to validate model performance. To apply the MCF-7−derived ML model to RPE cells, we used 25 features that were available in both MCF-7 and RPE cells. We used logistic regression with the L1 penalty to decide whether each intragenic cohesin site had decreased binding (1) or not (0). Then the MCF-7−fitted model was applied to the RPE features to predict DICs.

We also applied penalized regression followed by univariate linear regression as described^61^, to reveal which features contributed to negative or positive Mvalue (log ratio of cohesin peak intensity between E2 and control) in intragenic cohesin (26066 sites). We used 169 features (of the 175 features, 6 were excluded: 5 features related to cohesin position and the Mvalue feature) as independent variables ${X}_{i}=({X}_{i1},{X}_{i2},\ldots ,{X}_{{ij}})$, for $i=1,2,\ldots ,26066$ and $j=1,2,\ldots ,169$, whereas the Mvalue was the dependent variable ${Y}_{i}$. Instead of using the ordinary least squares approach, we used the elastic net loss function:

2 $$\begin{array}{c}{L}_{{enet}}\left(\hat{\beta }\right)=\frac{\mathop{\sum }\limits_{i=1}^{n}{\left({y}_{i}\;-\;{x}_{i}^{{\prime} }\hat{\beta }\right)}^{2}}{2n}+\lambda \left(\frac{1\,-\,\alpha }{2}\mathop{\sum }\limits_{j=1}^{169}{\hat{{\beta }_{j}}}^{2}+\alpha \mathop{\sum }\limits_{j=1}^{169}\left|\hat{{\beta }_{j}}\right|\right)\end{array}$$

to the linear model ${Y}_{i}={\beta }_{0}+\mathop{\sum }\nolimits_{j=1}^{169}{\beta }_{j}{X}_{{ij}}$, where $n=26066$ and $\hat{\beta }$ was the estimation of $\beta$. λ was chosen by cross-validation, and $\alpha =0.5$ was used to consider both the L1 and L2 penalty. Feature selection with such regularization was useful for filtering out non-significant or redundant features. The remaining features were applied to univariate linear regression $Y=a+{bX}$ to calculate the regression coefficient (Supplementary Figs. 9f–h).

### Extraction of DICs

Quantitative comparison of Rad21 binding events was performed using MAnorm^19^ version 1.3.0 with default parameters. This results in the normalized Mvalue, a quantitative measure of differential binding for all cohesin sites. To acquire more comprehensive cohesin binding sites, we combined our peak results with high-quality ChIP-seq data from E-TABM-828^6^. We excluded the cohesin sites with peaks width >3 kb and selected decreased peaks as M value < −0.5. Next, we used RefSeq genome annotations as the reference to obtain intragenic regions. As described in Supplementary Fig. 1e, we excluded 10 kb flanking regions around TSS and TES. Only large genes (gene length > 20 kb) were considered. E2-responsive genes were defined as genes with an increased Pol2ser2 ChIP-seq signal (ratio > 1.2) in the presence of E2 relative to control and that were validated by RNA-seq data. Decreased peaks at the intragenic region of 499 E2-responsive genes were defined as DICs. Next, to quantify CTCF read density on DICs, we used MULTICI options in DROMPA software. Peaks with very low Rad21 signals were omitted. Finally, we separated the DICs into 141 high-CTCF DICs (HC-DICs) and 417 low-CTCF DICs (LC-DICs).

### Motif analysis

Motifs were analyzed using HOMER. Briefly, peak files in standard bed format were converted to HOMER peak files, and then the command *findMotifsGenome.pl* was used to discover the motif. The results included known motifs as well as de novo discovered motifs. The size of the region used for motif finding was set to 200 bp. The top 10 motifs with the lowest *q* values (Benjamini-Hochberg) are shown.

### Software environment

All analyses were based on Ubuntu 18.04.4 with Python 3.6.9 and R 3.6.3. Data were processed using R base package or Numpy (v 1.17.2) as well as Pandas (v 0.25.1) in Python. Figures were drawn with DROMPA (v 3.7.2), Matplotlib (v 3.1.1), ggplot2 and R base plotting.

### Reporting summary

Further information on research design is available in the Nature Research Reporting Summary linked to this article.

## Supplementary information

Supplementary Information

Description of Additional Supplementary Files

Supplementary Data 1

Supplementary Data 2

Reporting Summary

## Data Availability

The raw sequencing data and processed files (peak files in bed format) have been deposited in the Gene Expression Ominibus (GEO) database under the series accession number GSE177045. The public Hi-C data for control and E2 treatment is available at GSE99541. The public H3K4me3, H3K27ac, H3K9ac, H3K14ac, H3K27me3, H3K9me3 ChIP-seq data for control and E2 treated MCF-7 cells are available at GSE23701. Public H3K4me1 ChIP-seq data are available at GSE40129. Public Rad21 ChIP-seq data are available at E-TABM-828. Public GRO-seq data are available at GSE99508. The human genome reference data used in this study is available at Ensembl (http://asia.ensembl.org/Homo_sapiens/Info/Index). The Fantom5 enhancer data is available at https://fantom.gsc.riken.jp/data/. Other public datasets used in this study are listed at Supplementary Tables 1–2 with the GEO database accession numbers. Source data are provided with this paper.

## Source data

Source Data
