# Supplementary material for: Large-scale multi-omics analysis suggests specific roles for intragenic cohesin in transcriptional regulation
Source: Nat Commun. 2022 Jun 9;13:3218. doi: 10.1038/s41467-022-30792-9 (PMC9184728; doi:10.1038/s41467-022-30792-9)
Supplement: Supplementary file 1 — Supplementary Information [file 41467_2022_30792_MOESM1_ESM.pdf]

Large-scale multi-omics analysis suggests specific roles for intragenic cohesin in transcriptional regulation

Jiankang Wang[1,2], Masashige Bando[1], Katsuhiko Shirahige[1,2], Ryuichiro Nakato[1,2,*]
[1]Institute for Quantitative Biosciences, The University of Tokyo; [2]Graduate School of Medicine, The University of Tokyo

Supplementary Figures 1-12

Supplementary Tables 1-4

Supplementary Figure 1

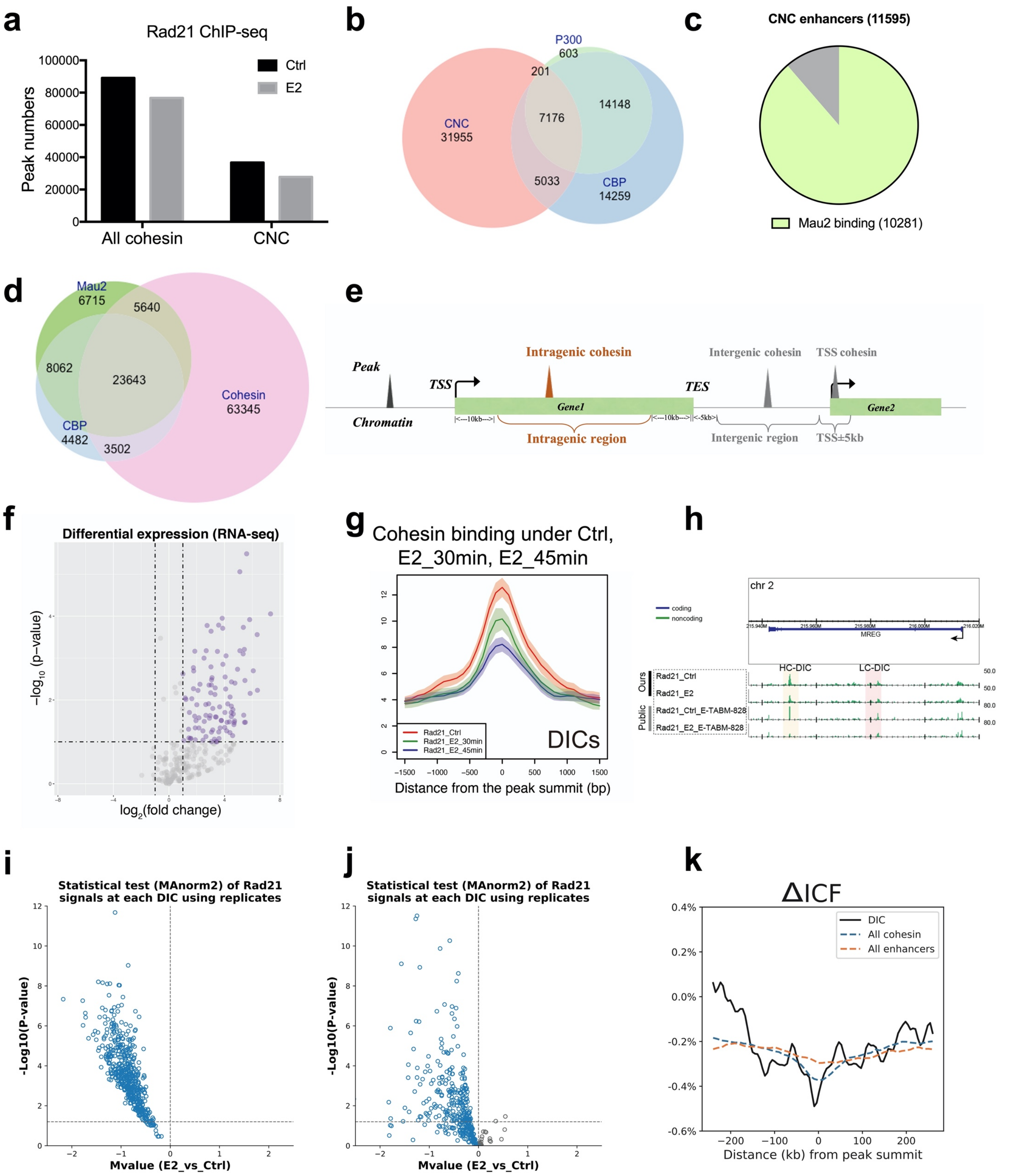

Supplementary Fig 1. ChIP-seq in MCF7 cells. a. Number of peaks for all cohesin (Rad21) and CNC (cohesin-non-CTCF) in control (Ctrl) and E2-treated MCF7 cells. **b.** Overlap among CNC, P300 and CBP sites. **c.** Among the CNC enhancer sites, most co-bound with Mau2. (CNC sites that also bound CBP were defined as CNC enhancers.) **d.** Overlap among Mau2, CBP and cohesin sites. **e.**The definition of intragenic regions for our analysis. TSS, transcription start site; TES, transcription end site. **f.** Differential expression of E2-responsive

genes between control and E2 treatment. Dashed lines represent $|\log_2(\text{fold change})| > 1$ and p-value < 0.05 (statistical tests implemented in DESeq2). **g.** E2 treatment for 30min indicated the gradually decreased cohesin binding on DICs. Shaded regions indicate 95% confidence intervals. **h**. Public data shows the similar decrease of binding levels on LC-DIC and HC-DIC sites. **i.** Statistical comparison (statistical tests implemented in MAnorm2) showed the deceasing tendency of each DIC. **j.** Statistical comparison (statistical tests implemented in MAnorm2) on the public Rad21 ChIP-seq data. **k.** Chromatin compaction scores around DICs, all cohesin sites and all enhancer sites (summit ± 500 kb). ΔICF (E2 vs. control) was calculated with 25-kb resolution.

Supplementary Figure 2

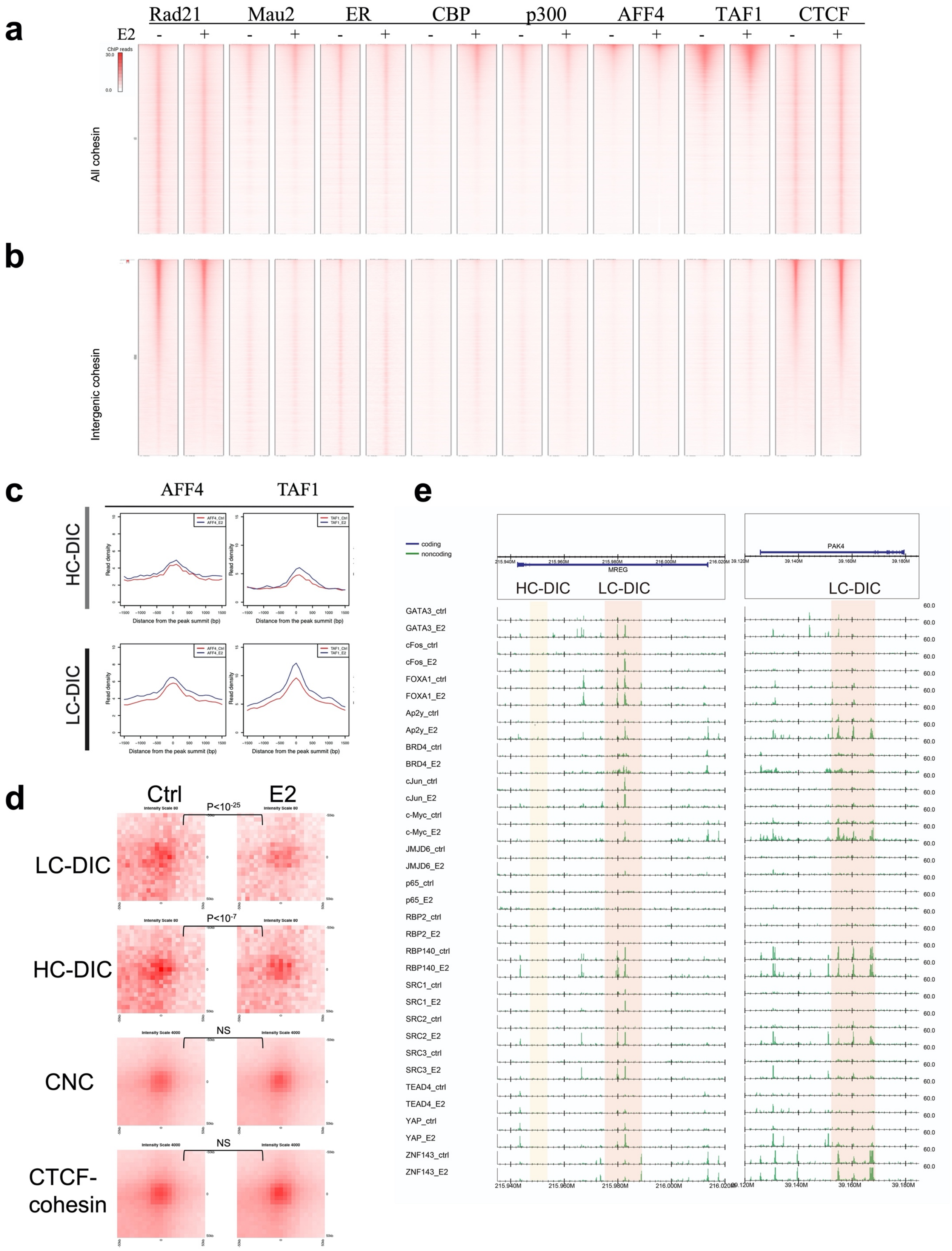

Supplementary Fig 2. Classification of LC-DIC and HC-DIC. a-b. Heatmap of ChIP-seq signals on (**a**) all cohesin and (**b**) intergenic cohesin sites (peak summit ± 2.5 kb), sorted by CTCF read density. **c.** Average

binding profiles for AFF4 and TAF1 on LC- and HC-DICs. **d.** APA plot shows the strength of chromatin interactions on LC-DICs ($p=1.0\times10^{-26}$), HC-DICs ($p=2.6\times10^{-8}$), CNC ($p=0.24$) and CTCF-cohesin ($p=0.39$). Comparisons (two-sided t-test) were conducted between E2 and Ctrl sample. No multiple testing adjustment was used. **e.** Publicly available data for TF binding to the MREG and PAK4 loci.

Supplementary Figure 3

a

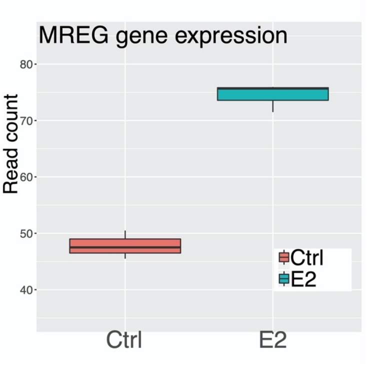

b

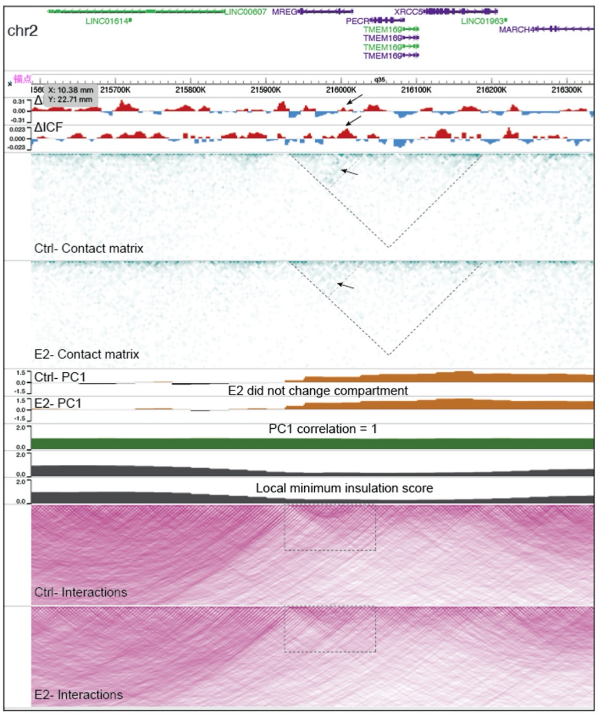

c

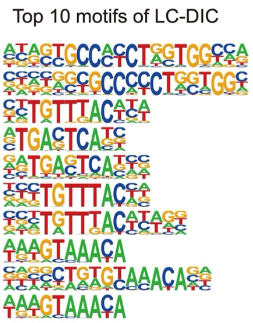

d

Motif Rank	LC-DIC	HC-DIC	Intragenic Cohesin	TSS cohesin	Intergenic cohesin	CNC	CTCF-cohesin
1	CTCF	CTCF	CTCF	CTCF	CTCF	CTCF	CTCF
2	BORIS	BORIS	BORIS	BORIS	BORIS	BORIS	BORIS
3	Foxa2	HiC1	Zic3	SCL	SCL	Fox	Tgif2
4	AP-1	Sox9	Unknown	Tgif2	NeuroD1	Foxa2	HIC1
5	Fosl2	NeuroD1	SCL	GRHL2	Zic3	FOXM1	SCL
6	Foxp1	SCL	HIC1	Fra2	Unknown	Foxa3	Zic3
7	Foxa3	ERE	NeuroD1	Fra1	Asc1	FOXA1	NeuroD1
8	Foxa1	Unknown	Tgif2	Fox:Ebox	Tgif2	AP2gamma	Unknown
9	Fox:Ebox	Tcf3	Zic2	BMYB	Unknown	AP2alpha	Ascl1
10	Foxk1	LEF1	Foxa2	Zic3	Tcf12	GRHL2	EBF1

Supplementary Fig 3. DICs were associated with active transcription, chromatin de-compaction and unique motifs. a. Expression of *MREG* from RNA-seq data (n=2 biological replicates). Box plots indicate the interquartile range IQR (25-75%) with a line at the median. Whiskers indicate 1.5 times the IQR. **b.** Chromatin organization near MREG gene. Each panel shows different features. Black arrows indicate DICs. Dashed triangles indicate a possible TAD and sub-TAD. Dashed boxes indicate the loss of interactions. **c.** Top 10 motif logos of LC-DICs. **d.** Top 10 motifs of LC-DICs, HC-DICs, intragenic cohesin, TSS cohesin, intergenic cohesin, CNC and CTCF-cohesin.

Supplementary Figure 4

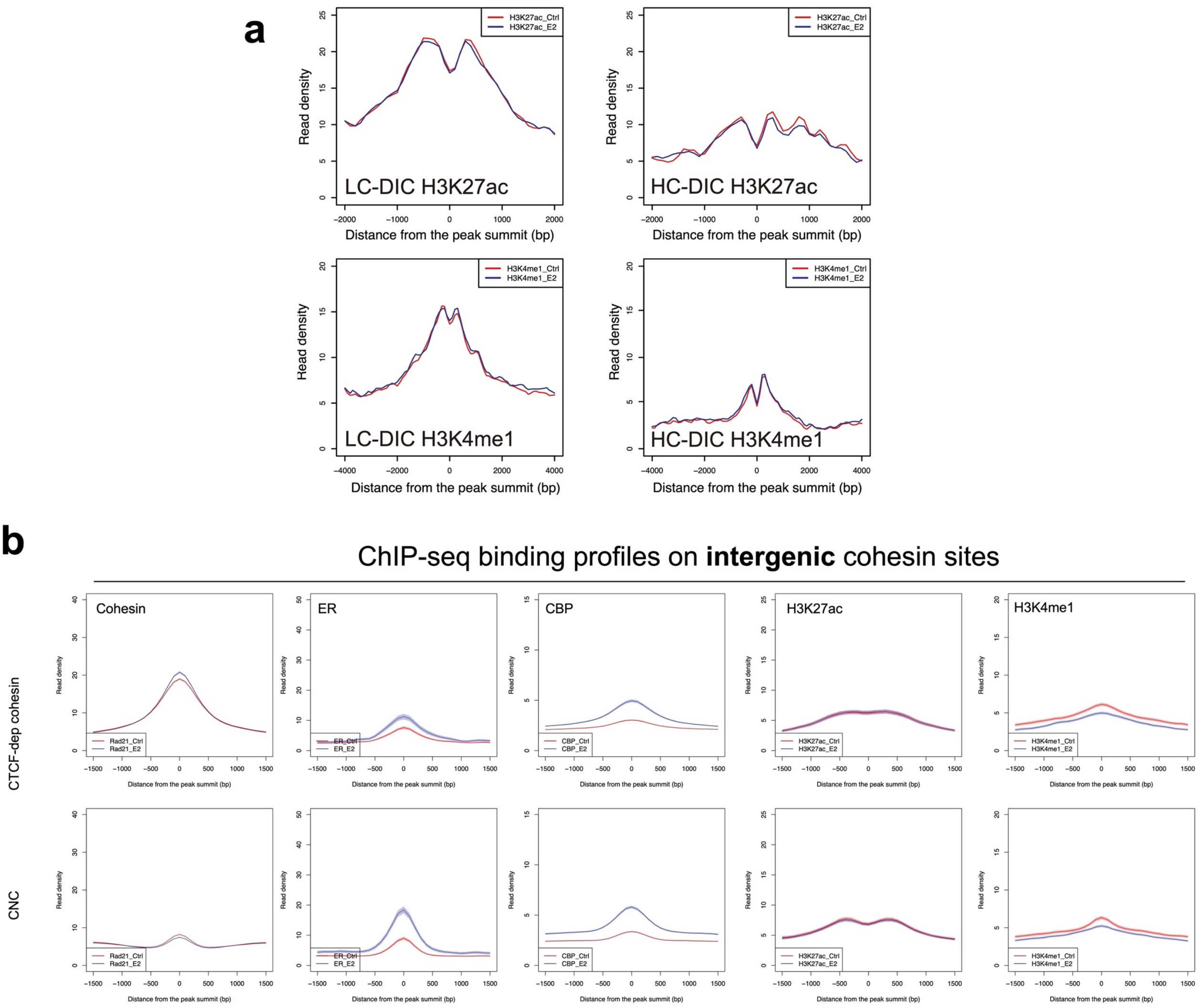

Supplementary Fig 4. Enhancer binding profiles on LC-DICs, HC-DICs and other cohesin sites. a. Binding profile (summit ± 2 kb) of enhancer markers around LC-DICs and HC-DICs, with Ctrl and E2 treatment. Reads were normalized relative to the whole genome. **b.** Binding profiles TFs and histone markers on CTCF-dependent (CTCF-dep) and CNC on intergenic regions (summit ± 1.5 kb). Shaded regions indicate 95% confidence intervals.

Supplementary Figure 5

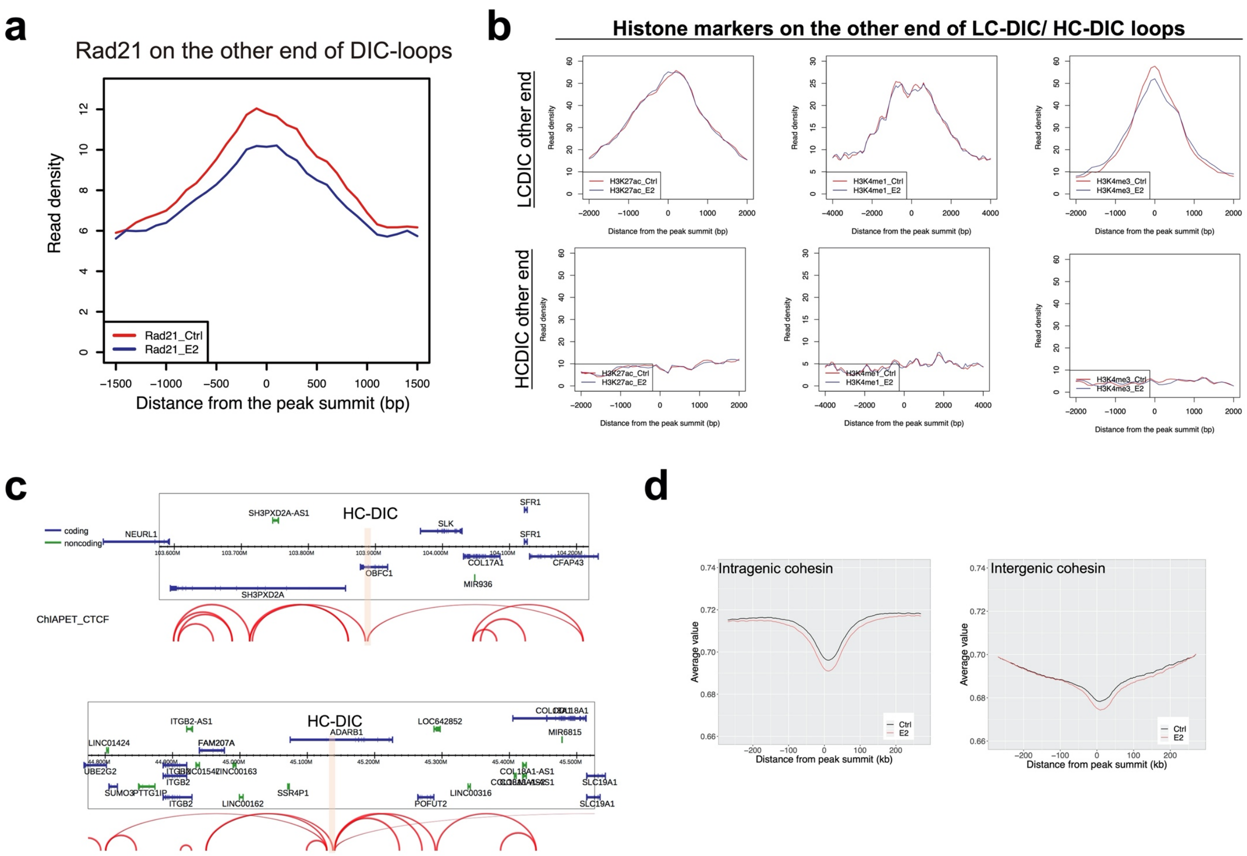

Supplementary Fig 5. DICs-anchored loops. a. Read density (summit ± 1.5 kb) of cohesin that interacted by DIC loops (i.e. the other end of DIC-loops). Such cohesin also showed a decreased binding profile after E2 treatment. **b.** Average binding profiles of histone modifications H3K27ac, H3K4me1 and H3K4me3 on the other end of LC-DIC/HC-DIC loops. **c.** Examples in which the other ends of the HC-DIC loops are in the introns of other genes. **d.** Insulation scores for all intragenic and intergenic cohesin sites.

Supplementary Figure 6

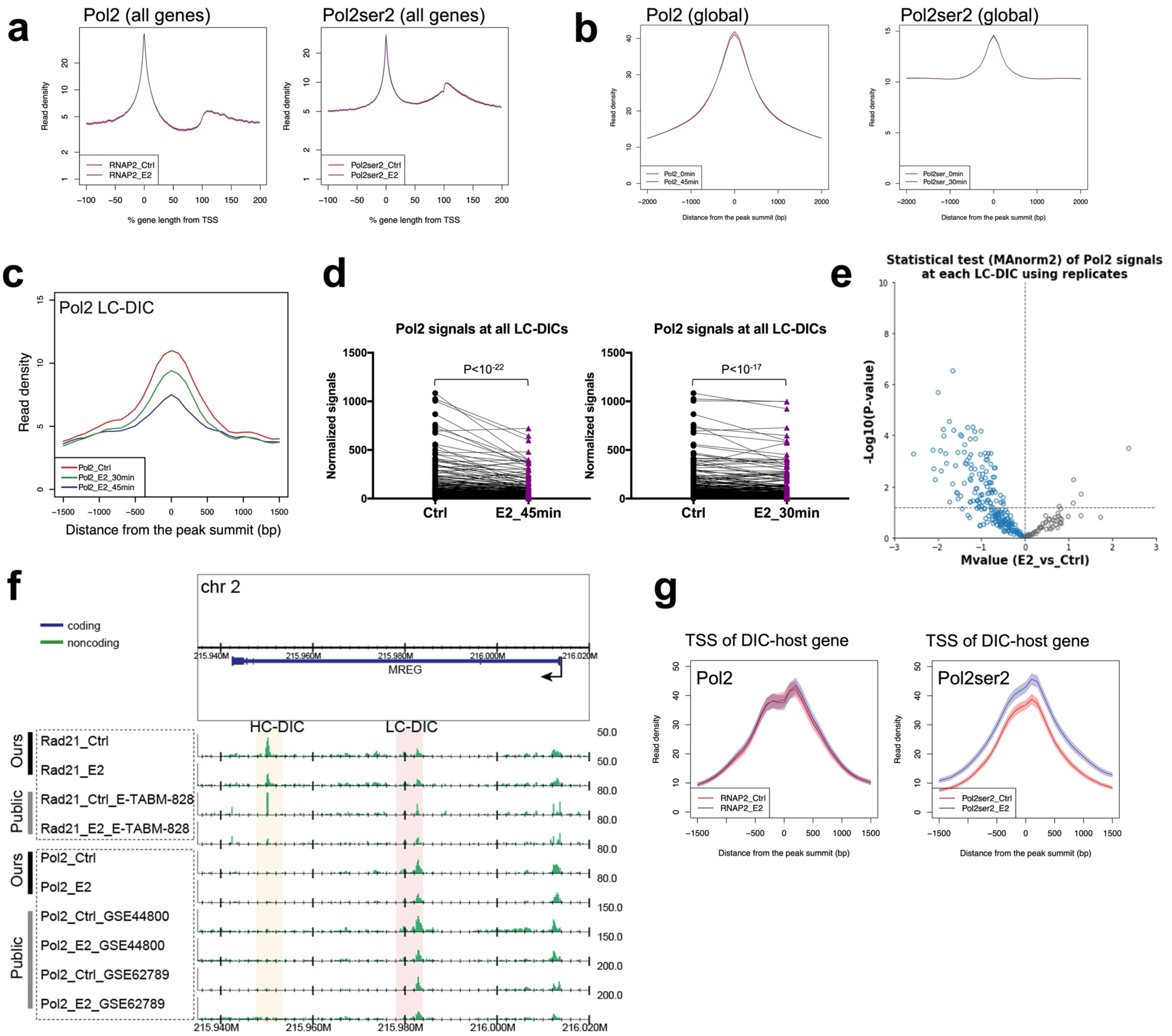

Supplementary Fig 6. Pol2 pausing on DICs. a. Read density of Pol2 and Pol2ser2 around all genes in Ctrl and E2-treated cells. **b.** Global read density of Pol2 and Pol2ser2 under Ctrl or E2 condition. **c.** E2 treatment for 30min showed the gradually decrease of Pol2 binding on DICs. **d.** Paired Wilcoxon-rank test (two-sided,n=417) showed the decreasing of Pol2 bindings at all LC-DIC sites for E2_45min ($p=6.1\times10^{-23}$) and E2_30min ($p=5.9\times10^{-18}$). **e.** Statistical comparison (statistical tests implemented in MAnorm2) showed the deceasing tendency of Pol2 at each DIC. **f.** Visualization of our ChIP-seq data and publicly available ChIP-seq were used to confirm the decrease in cohesin binding and the reduction of Pol2 on DIC sites. **g.** Average genomic binding profiles for Pol2 and Pol2ser2 around TSSs (±1.5 kb) of E2-responsive genes. Shaded regions indicate 95% confidence intervals.

Supplementary Figure 7

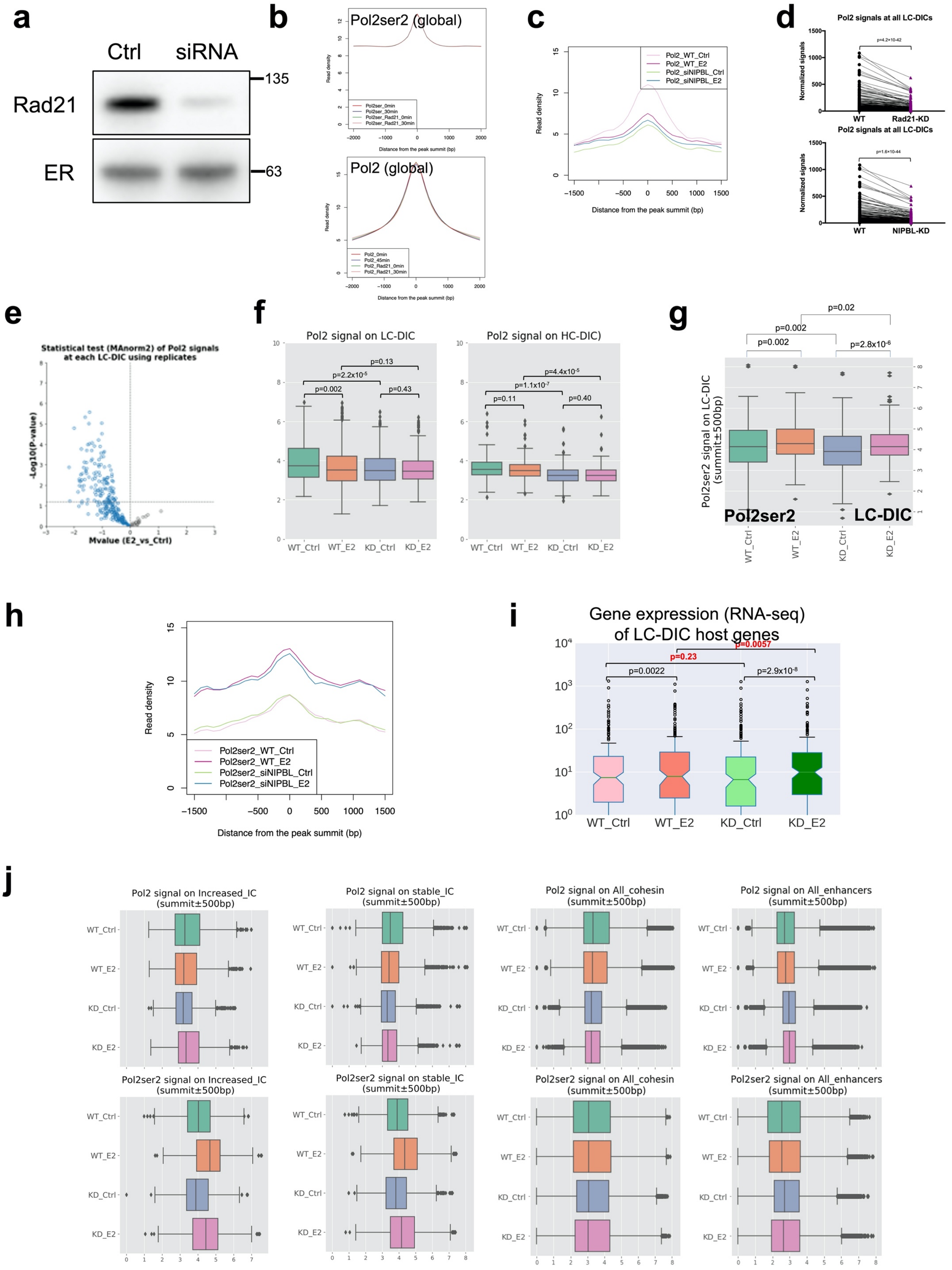

Supplementary Fig 7. Pol2 ChIP-seq under cohesin knockdown. a. Western blot of Rad21 in control and Rad21-knockdown MCF7 cells. The experiment was independently repeated three times with similar results. Source images are provided in the Source Data file. **b.** Global read density of Pol2 and Pol2ser2 under four different conditions. **c.** cohesin knockdown via NIPBL showed similar decreased Pol2. **d.** Paired Wilcoxon-rank test (two-sided, n=417) showed the decreasing of Pol2 bindings after Rad21-KD ($p=4.2\times10^{-42}$) or NIPBL-KD ($p=1.6\times10^{-44}$). **e.** Statistical comparison (statistical tests implemented in MAnorm2) showed the deceasing tendency of Pol2 after cohesin knockdown. **f.** Pol2 signal under four condition at LC- (n=417) or HC-DICs (n=141). Box plots indicate the interquartile range IQR (25-75%) with a line at the median. Whiskers indicate 1.5 times the IQR. Two-sided Mann–Whitney U-test was used. Multiple testing correlation with Benjamini-Hochberg method was used. Hereafter the black diamond symbols represent outliers. **g**. Replicate for Pol2ser2 at four conditions (n=417). Box plots indicate the interquartile range IQR (25-75%) with a line at the median. Whiskers indicate 1.5 times the IQR. Two-sided Mann–Whitney U-test was used. Two-sided Mann–Whitney U-test was used. Multiple testing correlation with Benjamini-Hochberg method was used. **h**. Pol2ser2 ChIP-seq with NIPBL knockdown. **i.** Expression of DIC host genes based on RNA-seq of MCF7 cells under the four conditions shown here. Red p value (Wilcox rank test, two-sided, n=198) indicated the comparison between KD and WT cells. Box plots indicate the interquartile range IQR (25-75%) with a line at the median. Whiskers indicate 1.5 times the IQR. **j.** Quantitative comparison of Pol2 and Pol2ser2 signals for various cohesin sites, including Increased_IC (increased intragenic cohesin, n=680); stable_IC (unchanged intragenic cohesin, n=2800); all cohesin (n=96218) and all enhancers (n=63285). Regions were restricted to the peak summit ± 500 bp. Box plots indicate the interquartile range IQR (25-75%) with a line at the median. Whiskers indicate 1.5 times the IQR.

Supplementary Figure 8

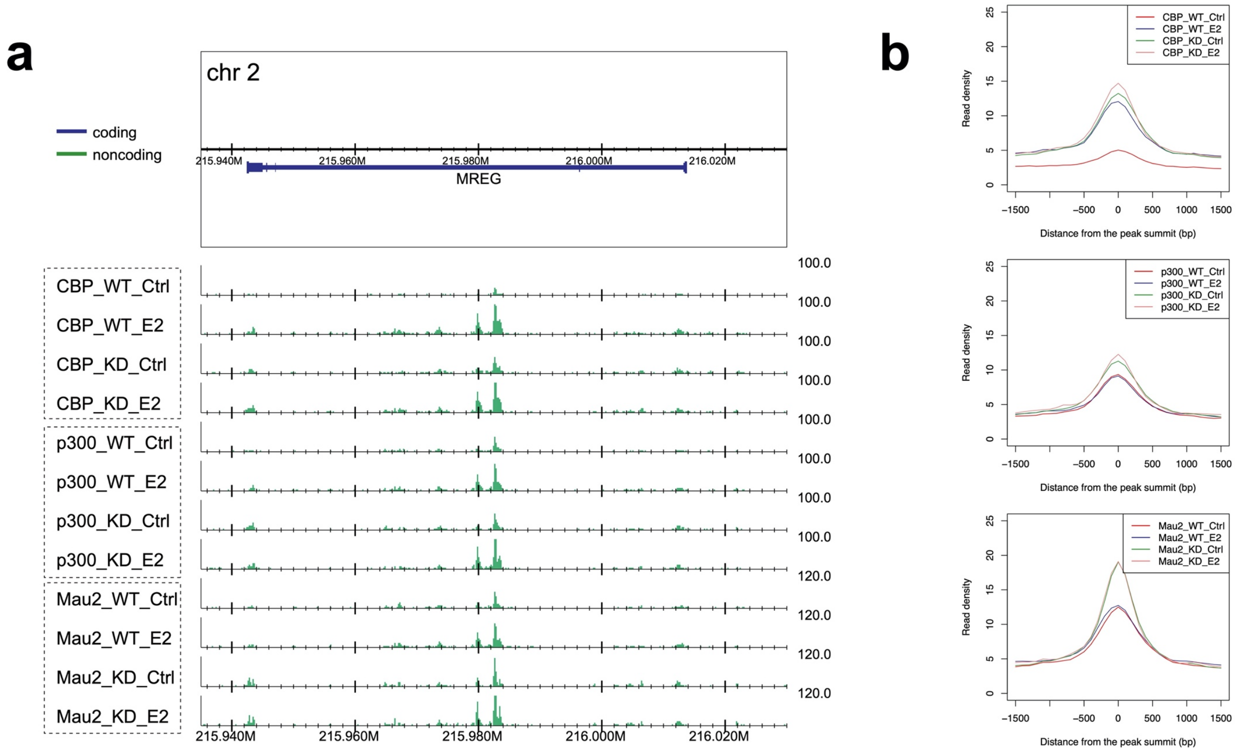

Supplementary Fig 8. Increased TFs after cohesin-knockdown on DICs. a. MREG locus showed the increased CBP, P300 and Mau2 after cohesin knockdown. **b.** Binding profile of CBP, P300 and Mau2 on LC-DICs under four different conditions as indicated.

Supplementary Figure 9

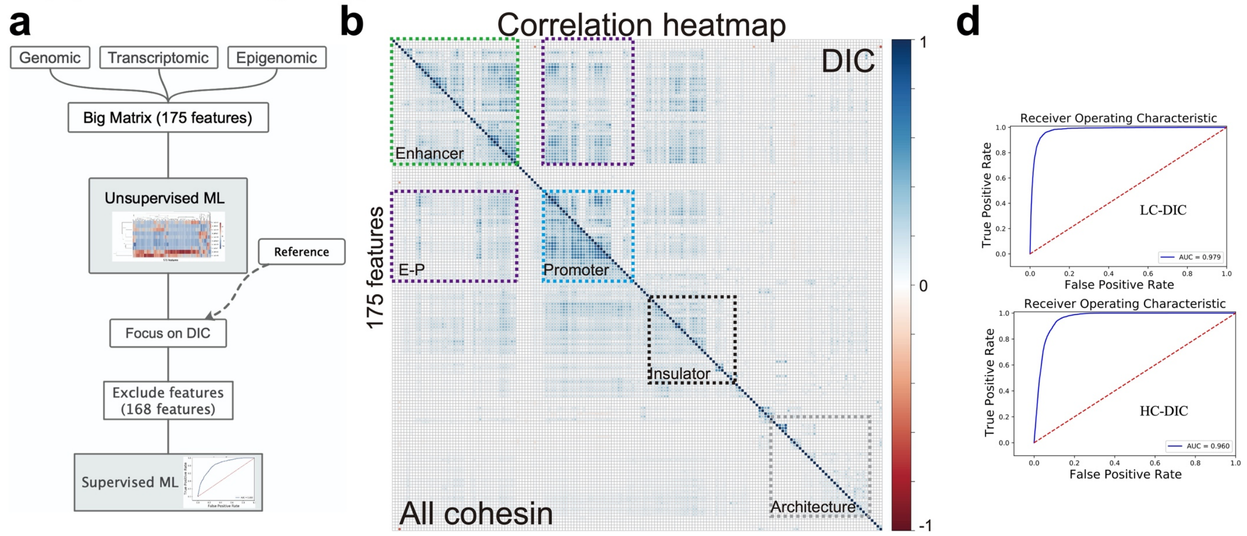

c

LC-DIC

Supervised method	Accuracy	Recall	F1 score	ROC
Logistic regression	0.941	0.920	0.708	0.969
SVM	0.905	0.960	0.495	0.968
Random Forest	0.975	0.687	0.735	0.980

HC-DIC

Supervised method	Accuracy	Recall	F1 score	ROC
Logistic regression	0.896	0.927	0.699	0.960
SVM	0.878	0.948	0.665	0.949
Random Forest	0.931	0.764	0.741	0.971

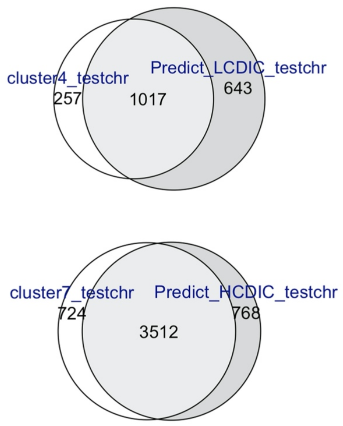

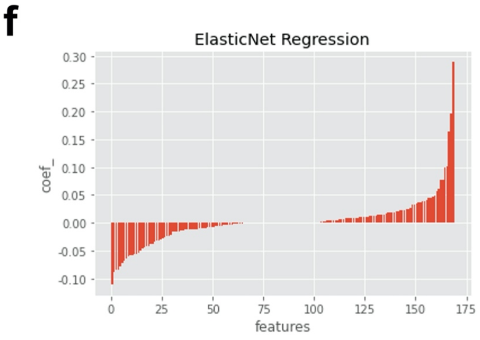

g

Features	Adjust-R2	coefficient
DNase	0.035	-0.177
FAIRE	0.072	-0.224
FOXA1	0.008	-0.086
GATA3	0.003	-0.059
H3K27ac	0.001	-0.036
MEN1	0.013	-0.132
Mau2	0.011	-0.094
Pol2	0.002	-0.049
p300	0.003	-0.067

h

Features	Adjust-R2	coefficient
ER	0.008	0.097
E2responseGene	0.011	0.130
H3K14ac	0.017	0.206
H3K27me3	0.002	0.123
H3K4me1	0.030	0.162
JMJD6	0.007	0.622
MED12	0.009	0.251
SRC1	0.011	0.189
SRC2	0.017	0.152

Supplementary Fig 9. Machine learning for DICs. a. Workflow for supervised and unsupervised machine learning. **b.** Correlation heatmap with hierarchical clustering of all 175 features for DICs (top right triangle) or all cohesin sites (bottom left triangle). The dashed boxes show different clusters of features. E-P, enhancer-promoter interactions. **c.** Performance score for supervised learning for LC-DICs and HC-DICs. **d.** Receiver operating characteristic (ROC) curve generated by the logistic regression model. **e.** The overlaps of predicted sites and real sites on test chromosomes 16–22, based on logistic regression. **f-h.** We analyzed all intragenic cohesin sites using penalty regression followed by univariate linear regression. The 169 features (6 features were excluded from the original 175 features: 5 features related to cohesin position and the Mvalue feature)

were independent variables, and Mvalue was the dependent variable. Feature selection by elastic net regression is shown (**f)**. We selected 58 features for the next univariate linear regression, and then we calculated the adjusted R^2 and regression coefficient between each selected feature and Mvalue. Features with negative coefficients (adjusted $R^2 > 0.001$) are shown in (**g**), and features with positive coefficients are shown in (**h**).

Supplementary Figure 10

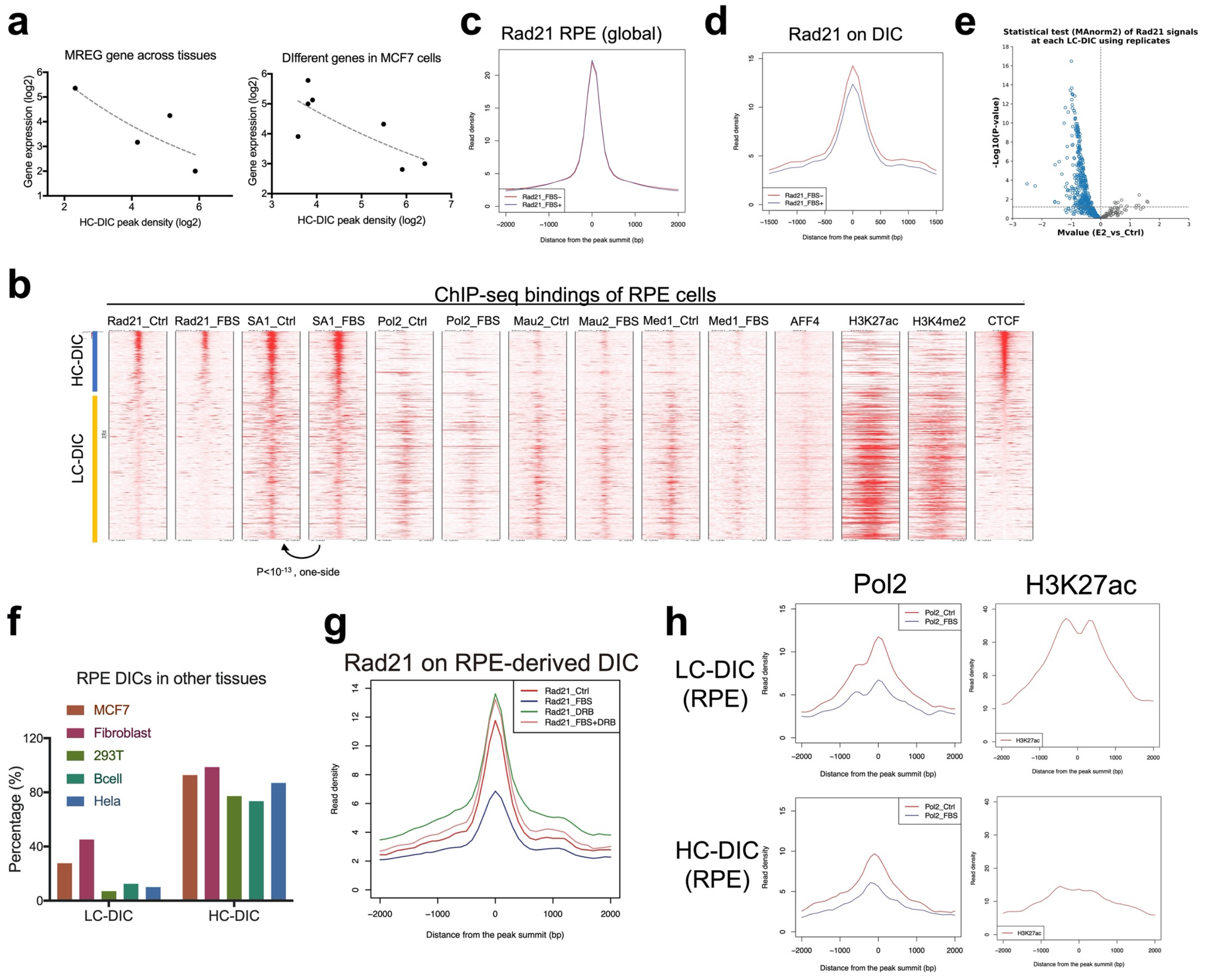

Supplementary Fig 10. DICs in other cell types. a. The relationships between HC-DIC peak density and the expression of genes that host HC-DICs. A negative correlation was observed across tissues (upper) and genes (lower). **b.** Heatmap of ChIP-seq reads at DICs of RPE cells (peak summit ± 2.5 kb). One-sided Wilcoxon signed-rank test was used. No multiple testing adjustment was used. The CTCF signal was used for sorting the order of DICs. **c.** Global read density of Rad21 under FBS-/+ in RPE cells. **d.** New replicates showed the similar decreasing of Rad21 bindings at DICs. **e.** Statistical comparison (statistical tests implemented in MAnorm2) showed the deceasing tendency of Rad21 after FBS treatment. **f.** The percentage of RPE DICs that could be found in other cell types. **g.** Rad21 binding profiles around RPE DICs (summit ± 2 kb) under four different conditions. **h.** ChIP-seq binding profiles of Pol2 and H3K27ac on RPE DICs.

Supplementary Figure 11

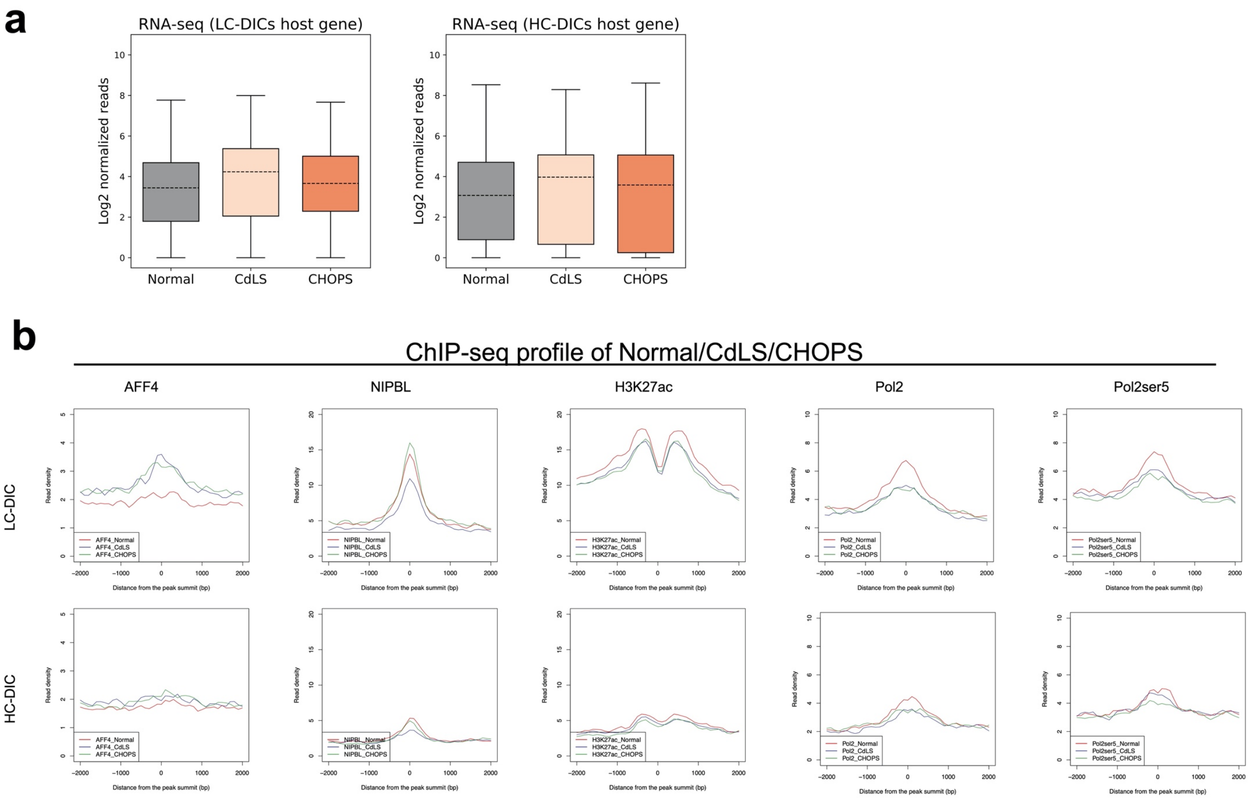

Supplementary Fig 11. DICs in CdLS and CHOPS. a. Expression of genes that host LC-DICs (n=185) and HC-DICs (n=147) in normal, CdLS and CHOPS cells. Box plots indicate the interquartile range IQR (25-75%) with a line at the median. Whiskers indicate 1.5 times the IQR. **b.** TF binding profiles on LC-DICs and HC-DICs. Red, normal; blue, CdLS; green, CHOPS.

Supplementary Fig 12. All clusters identified by K-means from Fig. 4. Box plots indicate the interquartile range IQR (25-75%) with a line at the mean. Whiskers indicate 95% confidence intervals.

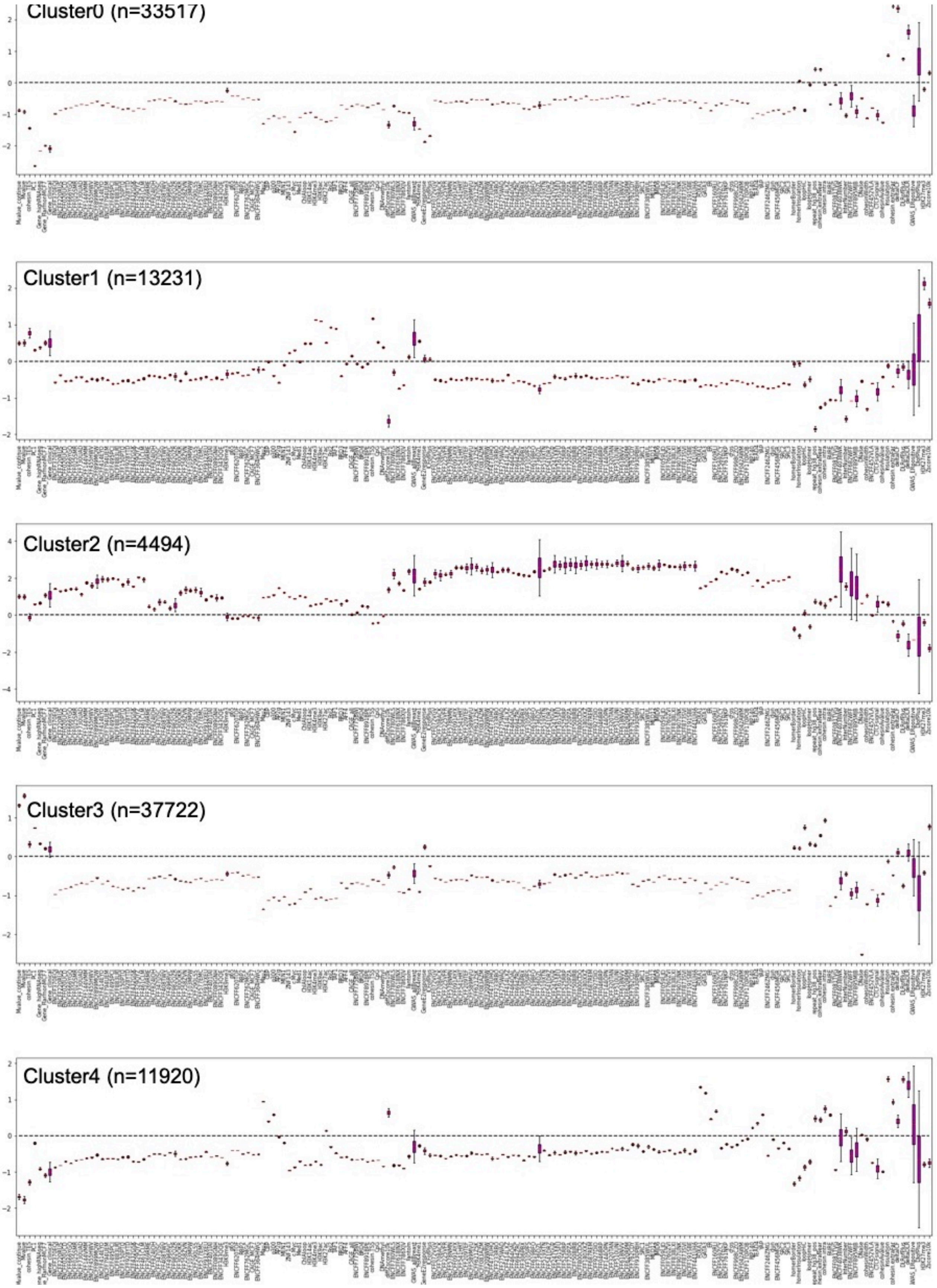

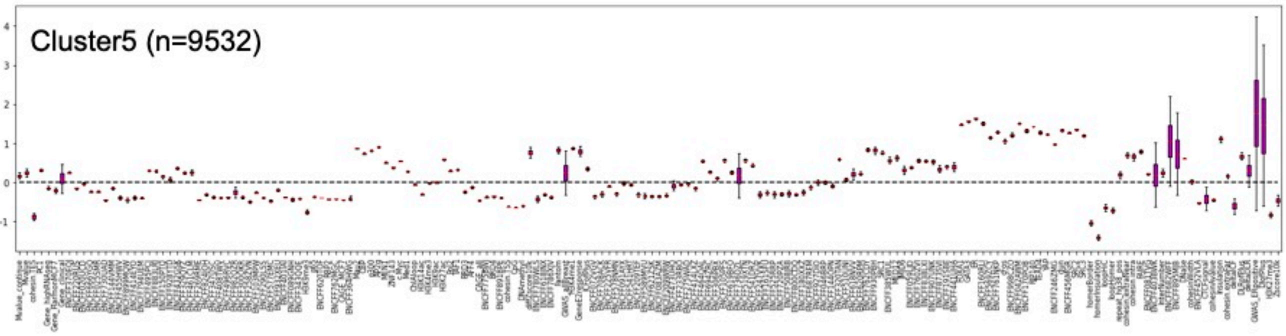
Cluster5 (n=9532)

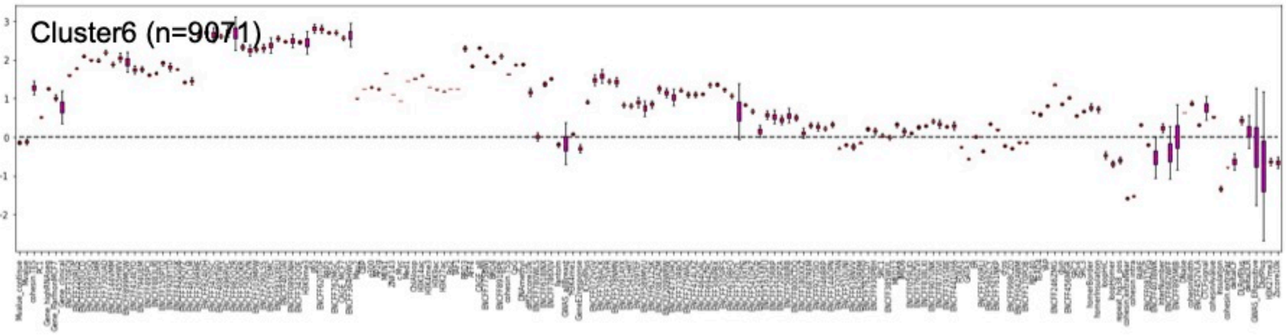
Cluster6 (n=9071)

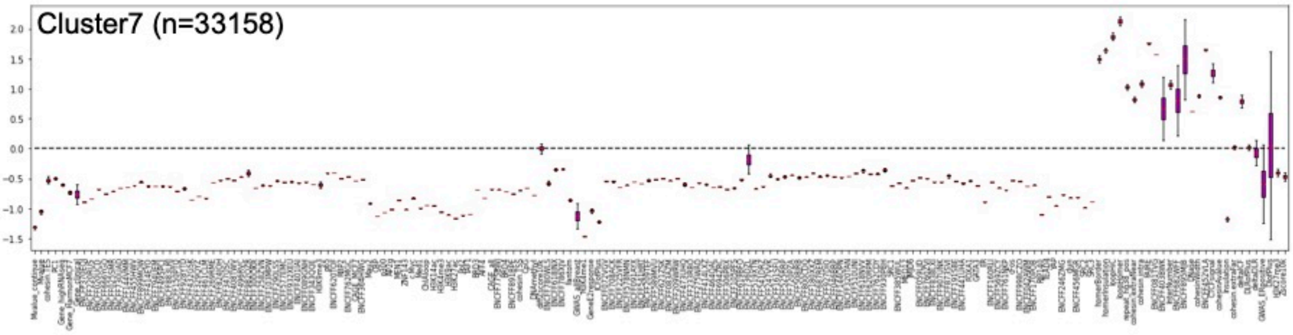
Cluster7 (n=33158)

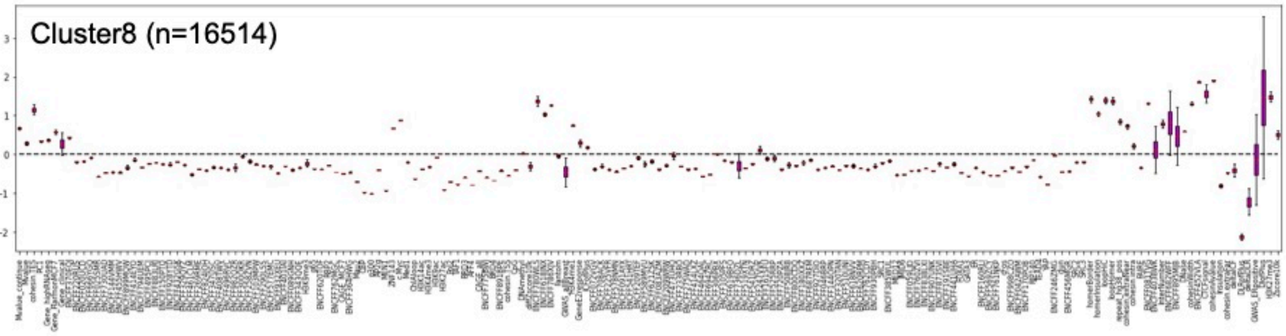
Cluster8 (n=16514)

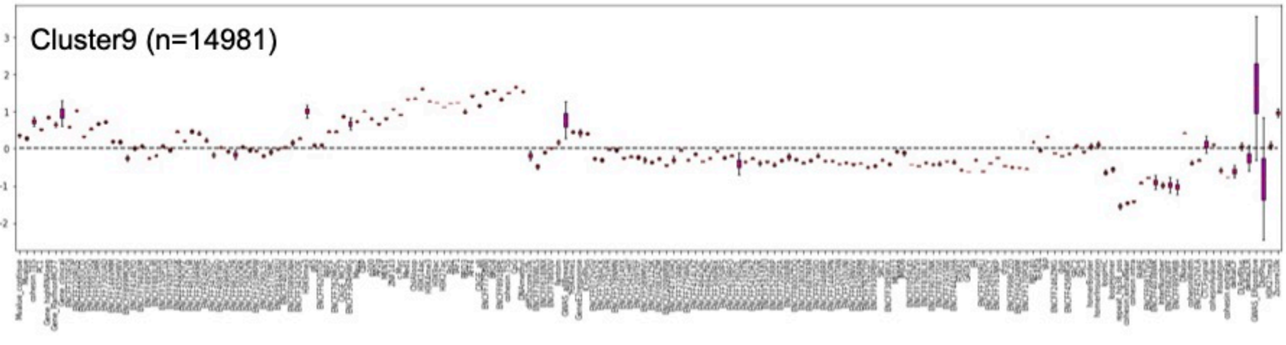
Cluster9 (n=14981)

Supplementary Tables

Supplementary Table 1:

Large scale ChIP-seq data used in this study. "This study" means that data were prepared by our lab.

Antibody	Cell line	Type	Treatment	Data Source
Rad21	MCF7	Wild-type	Ctrl	This study
Rad21	MCF7	Wild-type	E2_30min	This study
Rad21	MCF7	Wild-type	E2_45min	This study
Rad21(2 replicates)	MCF7	Wild-type	Ctrl	E-TABM-828
Rad21(2 replicates)	MCF7	Wild-type	E2	E-TABM-828
CTCF	MCF7	Wild-type	Ctrl and E2	This study
Mau2	MCF7	Wild-type	Ctrl and E2	This study
CBP	MCF7	Wild-type	Ctrl and E2	This study
p300	MCF7	Wild-type	Ctrl and E2	This study
ER	MCF7	Wild-type	Ctrl and E2	This study
Pol2	MCF7	Wild-type	Ctrl_rep1	This study
Pol2	MCF7	Wild-type	Ctrl_rep2	This study
Pol2	MCF7	Wild-type	E2_rep1	This study
Pol2	MCF7	Wild-type	E2_rep2	This study
Pol2	MCF7	Wild-type	E2_rep3	This study
Pol2	MCF7	Wild-type	Ctrl and E2	GSE62789
Pol2	MCF7	Wild-type	Ctrl and E2	GSE44800
Pol2ser2	MCF7	Wild-type	Ctrl_rep1	This study
Pol2ser2	MCF7	Wild-type	Ctrl_rep2	This study
Pol2ser2	MCF7	Wild-type	E2_rep1	This study
Pol2ser2	MCF7	Wild-type	E2_rep2	This study
TAF1	MCF7	Wild-type	Ctrl and E2	This study
AFF4	MCF7	Wild-type	Ctrl and E2	This study
H3K4me1	MCF7	Wild-type	Ctrl and E2	GSE40129
H3K4me3	MCF7	Wild-type	Ctrl and E2	GSE23701
H3K27ac	MCF7	Wild-type	Ctrl and E2	GSE23701
H3K9ac	MCF7	Wild-type	Ctrl and E2	GSE23701
H3K14ac	MCF7	Wild-type	Ctrl and E2	GSE23701
H3K27me3	MCF7	Wild-type	Ctrl and E2	GSE23701
H3K9me3	MCF7	Wild-type	Ctrl and E2	GSE23701
Pol2 (Rad21KD)	MCF7	Cohesin-KD	KD_Ctrl_rep1	This study
Pol2 (Rad21KD)	MCF7	Cohesin-KD	KD_Ctrl_rep2	This study
Pol2 (Rad21KD)	MCF7	Cohesin-KD	KD_E2_rep1	This study
Pol2 (Rad21KD)	MCF7	Cohesin-KD	KD_E2_rep2	This study
Pol2 (NIPBLKD)	MCF7	Cohesin-KD	Ctrl	This study
Pol2 (NIPBLKD)	MCF7	Cohesin-KD	E2	This study

Pol2ser2	MCF7	Cohesin-KD	KD_Ctrl_rep1	This study
Pol2ser2	MCF7	Cohesin-KD	KD_Ctrl_rep2	This study
Pol2ser2	MCF7	Cohesin-KD	KD_E2_rep1	This study
Pol2ser2	MCF7	Cohesin-KD	KD_E2_rep2	This study
Mau2	MCF7	Cohesin-KD	Ctrl and E2	This study
CBP	MCF7	Cohesin-KD	Ctrl and E2	This study
p300	MCF7	Cohesin-KD	Ctrl and E2	This study
c-Fos	MCF7	Wild-type	Ctrl and E2	GSE26831
FOXA1	MCF7	Wild-type	Ctrl and E2	GSE112969
GATA3	MCF7	Wild-type	Ctrl and E2	GSE40129
Input	MCF7	Wild-type	Ctrl and E2	This study
Rad21	B-cell	Wild-type	None	This study
Rad21	Fibroblast	Wild-type	None	This study
Rad21	HeLa	Wild-type	None	This study
Rad21	RPE	Wild-type	None	This study
Rad21	293T	Wild-type	None	This study
Rad21	RPE	Wild-type	FBS –_rep1	This study
Rad21	RPE	Wild-type	FBS –_rep2	This study
Rad21	RPE	Wild-type	FBS +_rep1	This study
Rad21	RPE	Wild-type	FBS +_rep2	This study
Rad21	RPE	Wild-type	DRB – and +	This study
Rad21	RPE	Wild-type	FBS+DRB+	This study
SA1	RPE	Wild-type	FBS – and +	This study
POL2	RPE	Wild-type	FBS – and +	This study
RNA Pol2ser2	RPE	Wild-type	FBS – and +	This study
Mau2	RPE	Wild-type	FBS – and +	This study
Med1	RPE	Wild-type	FBS – and +	This study
H3K27ac	RPE	Wild-type	FBS – and +	This study
Input	RPE	Wild-type	FBS – and +	This study
Input	RPE	Wild-type	DRB – and +	This study
Input	RPE	Wild-type	FBS+ and DRB+	This study
Rad21	Fibroblast	Normal	None	This study
Rad21	Fibroblast	CdLS	None	This study
Rad21	Fibroblast	CHOPS	None	This study
NIPBL	Fibroblast	Normal	None	This study
NIPBL	Fibroblast	CdLS	None	This study
NIPBL	Fibroblast	CHOPS	None	This study
AFF4	Fibroblast	Normal	None	This study
AFF4	Fibroblast	CdLS	None	This study
AFF4	Fibroblast	CHOPS	None	This study

H3K27ac	Fibroblast	Normal	None	This study
H3K27ac	Fibroblast	CdLS	None	This study
H3K27ac	Fibroblast	CHOPS	None	This study
Pol2	Fibroblast	Normal	None	This study
Pol2	Fibroblast	CdLS	None	This study
Pol2	Fibroblast	CHOPS	None	This study
Pol2ser5	Fibroblast	Normal	None	This study
Pol2ser5	Fibroblast	CdLS	None	This study
Pol2ser5	Fibroblast	CHOPS	None	This study
Input	Fibroblast	Normal	None	This study
Input	Fibroblast	CdLS	None	This study
Input	Fibroblast	CHOPS	None	This study

Supplementary Table 2:

Other omics datasets used in this study.

Omics	Cell line	Condition	Replicates	Source
Hi-C (in situ)	MCF7	Ctrl	2	GSE99541
Hi-C (in situ)	MCF7	E2	2	GSE99541
RNA-seq	MCF7	Ctrl	4	GSE89888
RNA-seq	MCF7	E2	4	GSE89888
GRO-seq	MCF7	Ctrl	2	GSE99508
GRO-seq	MCF7	E2	2	GSE99508
ChIA-PET Pol2	MCF7	none	2	GSE33664
ChIA-PET CTCF	MCF7	none	2	GSE39495
RNA-seq	Fibroblast	Normal	2	This study
RNA-seq	Fibroblast	CdLS	2	This study
RNA-seq	Fibroblast	CHOPS	2	This study
RNA-seq	MCF7	WT_Ctrl	2	This study
RNA-seq	MCF7	WT_E2	2	This study
RNA-seq	MCF7	KD_E2	2	This study
RNA-seq	MCF7	KD_E2	2	This study
FANTOM5	Enhancer dataset			
GTEx Portal	Genotype tissue expression			

Supplementary Table 3:

Changes of main TFs at LC-DIC sites. “up” means increased binding after E2 treatment, while “none” means no change.

TFs	Function	Change
FOXA1	Transcriptional activators	up
GATA3	GATA family of transcription factors	up
Ap2y	Activate protein 2 family of transcription factors	up
BRD3 / 4	Bromodomain-containing protein 3 and 4	up
c-Fos	Form transcription factor complex AP-1	up
c-Jun	Form AP-1 early response transcription factor	up
c-myc	Classical transcription factor	up
p65	NF-kappa-B p65 subunit	none
RBP2	Participate in the uptake and/or intracellular metabolism of vitamin A.	none
RIP140	Key regulator which modulates transcriptional activity of many TFs	up
SRC1/2/3	Transcriptional coactivator for steroid and nuclear hormone receptor	up
TEAD4	Transcriptional enhancer factor TEF-3	up
YAP	Transcriptional regulator by activating gene	Slightly up
ZNF143	Zinc Finger Protein 143	none

Supplementary Table 4:

175 Features (160 binomial and 15 continuous) extracted from multiomics data for machine learning. All those datasets were based on wild type MCF7 cell line.

Omics types	Data types (on each cohesin sites)	Feature types	Number	Source
Epigenome	ChIP-seq of Mau2, CBP, p300, Pol2, ER, AFF4, TAF1.	Binomial (0 or 1)	7	Our study
Epigenome	ChIP-seq of Ap2y, BRD3, BRD4, c-Fos, c-Jun, c-myc, FOXA1, GATA3, JMJD6, Med12, Med1, Men1, p65, RBP2, RIP140, SRC1/2/3, TEAD4, YAP, ZNF143.	Binomial (0 or 1)	21	GSE60270,109571,55921,26831,102410,33213,112969,40129,101559,60270,85317,59530,28337,125594,76460; E-MTAB-2576,785
Epigenome	ChIP-seq of H3K9ac, H3K14ac, H3K27ac, H3K4me1, H3K4me3, H3K27me3, H3K9me3	Binomial (0 or 1)	7	GSE23701
Epigenome	Peak width of cohesin	Continuous (log)	1	Our study
Epigenome	Peak intensity of cohesin (Avalue)	Continuous (log)	1	Our study
Epigenome	CTCF peak intensity	Continuous (log)	1	Our study
Others	Log ratio (E2 vs Ctrl) of cohesin peak intensity (Mvalue).	Continuous	1	Our study
Epigenome	FAIRE-seq and DNase-seq	Binomial (0 or 1)	2	Encode project[1]
Epigenome	ChIP-seq of more 103 TFs for MCF7 cells	Binomial (0 or 1)	103	Encode project
Epigenome	Amount of DNA methylation sites(RRBS-seq)	Continuous (log)	1	Encode project
Epigenome	Metrics of HiC, including insulation score, directionality index, DLR, ICF, deltaDLR, deltaICF, interaction number, zscore and	Continuous	10	GSE99541

1 https://www.encodeproject.org

	diffZscore from Homer software.			
Epigenome	Loop anchors, chromatin compartment, TAD border from HiC; Pol2 loop anchors from ChIA-PET.	Binomial (0 or 1)	4	GSE99541, GSE33664
Others	Enhancer sites or not	Binomial (0 or 1)	1	Fantom5 project[2]
Genome	CpG island	Binomial (0 or 1)	1	UCSC genome browser[3]
Genome	Repeat region	Binomial (0 or 1)	1	UCSC genome browser
Others	Chromatin location of cohesin: TSS, TES, extra-near-gene (gene±100kb), extra-far-gene, intragenic.	Binomial (0 or 1)	5	Our study
Transcriptome	TSS sites (CAGE-seq), for general cell lines, and MCF7 cells.	Binomial (0 or 1)	2	Fantom5 project
Transcriptome	Nearby E2-response genes, MCF highly expressed genes (RNAseq), MCF7 specific genes, breast cancer related genes	Binomial (0 or 1)	4	Our study, GSE89888, harmonizome database[4], PMID30234119
Others	GWAS: overlap with breast cancer SNPs; overlap with MCF7 cell SNPs.	Binomial (0 or 1)	2	GWAS Catalog database[5]

---

2 https://fantom.gsc.riken.jp/5/
3 https://genome.ucsc.edu
4 https://maayanlab.cloud/Harmonizome/
5 https://www.ebi.ac.uk/gwas/
