# Supplementary material for: Large-scale multi-omics analysis suggests specific roles for intragenic cohesin in transcriptional regulation
Source: Nat Commun. 2022 Jun 9;13:3218. doi: 10.1038/s41467-022-30792-9 (PMC9184728; doi:10.1038/s41467-022-30792-9)
Supplement: Supplementary file 2 — Description of Additional Supplementary Files [file 41467_2022_30792_MOESM2_ESM.pdf]

Description of Additional Supplementary Files

File Name: Supplementary Data 1
Description: Statistics and quality metrics of ChIP-seq data generated in this study.

File Name: Supplementary Data 2
Description: Statistics and quality metrics of RNA-seq data generated in this study.
