# Supplementary material for: Large-scale multi-omics analysis suggests specific roles for intragenic cohesin in transcriptional regulation
Source: Nat Commun. 2022 Jun 9;13:3218. doi: 10.1038/s41467-022-30792-9 (PMC9184728; doi:10.1038/s41467-022-30792-9)
Supplement: Supplementary file 5 — Reporting Summary [file 41467_2022_30792_MOESM5_ESM.pdf]

nature portfolio

Corresponding author(s): Nakato Ryuichiro

Last updated by author(s): May 12, 2022

Reporting Summary

Nature Portfolio wishes to improve the reproducibility of the work that we publish. This form provides structure for consistency and transparency in reporting. For further information on Nature Portfolio policies, see our Editorial Policies and the Editorial Policy Checklist.

Statistics

For all statistical analyses, confirm that the following items are present in the figure legend, table legend, main text, or Methods section.

n/a	Confirmed	
☐	☒	The exact sample size (*n*) for each experimental group/condition, given as a discrete number and unit of measurement
☐	☒	A statement on whether measurements were taken from distinct samples or whether the same sample was measured repeatedly
☐	☒	The statistical test(s) used AND whether they are one- or two-sided<br>*Only common tests should be described solely by name; describe more complex techniques in the Methods section.*
☒	☐	A description of all covariates tested
☐	☒	A description of any assumptions or corrections, such as tests of normality and adjustment for multiple comparisons
☐	☒	A full description of the statistical parameters including central tendency (e.g. means) or other basic estimates (e.g. regression coefficient) AND variation (e.g. standard deviation) or associated estimates of uncertainty (e.g. confidence intervals)
☐	☒	For null hypothesis testing, the test statistic (e.g. *F*, *t*, *r*) with confidence intervals, effect sizes, degrees of freedom and *P* value noted<br>*Give P values as exact values whenever suitable.*
☒	☐	For Bayesian analysis, information on the choice of priors and Markov chain Monte Carlo settings
☒	☐	For hierarchical and complex designs, identification of the appropriate level for tests and full reporting of outcomes
☐	☒	Estimates of effect sizes (e.g. Cohen's *d*, Pearson's *r*), indicating how they were calculated

Our web collection on statistics for biologists contains articles on many of the points above.

Software and code

Policy information about availability of computer code

Data collection	External data was acquired using fastq-dump SRA toolkit version 2.10.5 when raw files were uses. Otherwise no software was used.
Data analysis	Bowtie version 1.2.2; DROMPA version 3.7.2; MACS2 version 2.2.6; Bedtools version 2.29.2; Samtools version 1.9; Juicer version 1.11.04; HOMER version v4.11; Mango v1.2.0; HISAT2 version 2.2.0; Htseq version 0.11.3; DESeq2; scikit-learn version 0.22.1; MAnorm version 1.3.0;

For manuscripts utilizing custom algorithms or software that are central to the research but not yet described in published literature, software must be made available to editors and reviewers. We strongly encourage code deposition in a community repository (e.g. GitHub). See the Nature Portfolio guidelines for submitting code & software for further information.

Data

Policy information about availability of data

All manuscripts must include a data availability statement. This statement should provide the following information, where applicable:

- Accession codes, unique identifiers, or web links for publicly available datasets
- A description of any restrictions on data availability
- For clinical datasets or third party data, please ensure that the statement adheres to our policy

The raw sequencing data and processed files (peak files in bed format) have been deposited in the Gene Expression Ominibus (GEO) database under the series accession number GSE177045 [https://www.ncbi.nlm.nih.gov/geo/query/acc.cgi?acc=GSE177045]. The public Hi-C data for control and E2 treatment is available at GSE99541 [https://www.ncbi.nlm.nih.gov/geo/query/acc.cgi?acc=GSE99541]. The public H3K4me3, H3K27ac, H3K9ac, H3K14ac, H3K27me3, H3K9me3 ChIP-seq data for control and E2 treated MCF-7 cells are available at GSE23701 [https://www.ncbi.nlm.nih.gov/geo/query/acc.cgi?acc=GSE23701]. Public H3K4me1 ChIP-seq data are available at GSE40129 [https://www.ncbi.nlm.nih.gov/geo/query/acc.cgi?acc=GSE40129]. Public Rad21 ChIP-seq data are available at E-TABM-828 [https://www.ebi.ac.uk/arrayexpress/experiments/E-TABM-828/]. Public GRO-seq data are available at GSE99508 [https://www.ncbi.nlm.nih.gov/geo/query/acc.cgi?acc=

March 2021

GSE99508]. The human genome reference data used in this study is available at Ensembl [http://asia.ensembl.org/Homo_sapiens/Info/Index]. The Fantom5 enhancer data is available at https://fantom.gsc.riken.jp/data/. Other public datasets used in this study are listed at Supplementary Tables S1-2 with the GEO database accession numbers. Source data are provided with this paper.

Field-specific reporting

Please select the one below that is the best fit for your research. If you are not sure, read the appropriate sections before making your selection.

☒ Life sciences ☐ Behavioural & social sciences ☐ Ecological, evolutionary & environmental sciences

For a reference copy of the document with all sections, see nature.com/documents/nr-reporting-summary-flat.pdf

Life sciences study design

All studies must disclose on these points even when the disclosure is negative.

Sample size	No statistical method was used to predetermine sample size. We determined sample size according to common practice in the field and our previous knowledge about sample variation for the respective technique. Sample size selection was also based on availability (e.g., number of primary samples) and to provide sufficient statistical power to identify significant differences.
Data exclusions	No data were excluded from the analyses.
Replication	RNA-seq was performed in duplicates. The public Hi-C, GRO-seq, ChIA-PET data used in these study have duplicates. ChIP-seq was performed with duplicates. ChIP-seq was also confirmed by public data as described in manuscript. All replication status are also listed in Supplementary Tables 2-3.
Randomization	Randomization was not relevant to this study because we didn't have group allocation in the experiments.
Blinding	No blinding was performed because all the data of the study were analyzed without pre-established conclusions. The results of our experiments are obtained by objective quantitative methods.

Reporting for specific materials, systems and methods

We require information from authors about some types of materials, experimental systems and methods used in many studies. Here, indicate whether each material, system or method listed is relevant to your study. If you are not sure if a list item applies to your research, read the appropriate section before selecting a response.

Materials & experimental systems

n/a	Involved in the study	
☐	☒	Antibodies
☐	☒	Eukaryotic cell lines
☒	☐	Palaeontology and archaeology
☒	☐	Animals and other organisms
☒	☐	Human research participants
☒	☐	Clinical data
☒	☐	Dual use research of concern

Methods

n/a	Involved in the study	
☐	☒	ChIP-seq
☒	☐	Flow cytometry
☒	☐	MRI-based neuroimaging

Antibodies

Antibodies used	Rabbit polyclonal antibody for Rad21 (1:1000 dilution for western blot; 2.5 ug/million cells for ChIP-seq), which has been described in PMID:26051894, was obtained from Eurofins Genomics (peptides DEPIIEEPSR and ATPGPRFHII with affinity-purification). Antibodies for MAU2 (ab46906, 2.5 ug/million cells as dilution) and SA1 (ab4457, 2.5 ug/million cells as dilution) were from Abcam. Antibodies for TAF1 (A303-505A, 2.5 ug/million cells as dilution) and AFF4 (A302-538A, 2.5 ug/million cells as dilution) were from Bethyl Laboratory. CTCF (07-729, 2.5 ug/million cells as dilution) antibody was from Merck Millipore. Antibodies (2.5 ug/million cells as dilution) for unphosphorylated Pol2 (CMA601), Pol2ser2 (CMA602) and H3K27ac (CMA309) were kindly provided by Dr. H Kimura (Tokyo Institute of Technology), which were decribed in previous studies PMID: 25252976 and PMID: 25730767. Antibody for CBP (606402, 2.5 ug/million cells as dilution) was from BioLegend. Antibodies for P300 (sc-585, 2.5 ug/million cells as dilution) and Med1 (sc-5334, 2.5 ug/million cells as dilution) were from Santa Cruz Biotechnology.
Validation	The manufacturers or related reference provide the validation information of the antibodies:<br>Rad21: https://www.sciencedirect.com/science/article/pii/S0960982215005916?via%3Dihub<br>Mau2: https://www.abcam.co.jp/scc4-antibody-epr14390-ab183033.html<br>SA1: https://www.abcam.co.jp/sa1-antibody-ab4457.html<br>TAF1: https://www.fortislife.com/products/primary-antibodies/rabbit-anti-taf1-antibody/BETHYL-A303-505<br>AFF4: https://www.fortislife.com/products/primary-antibodies/rabbit-anti-mcef-antibody/BETHYL-A302-538<br>CTCF: https://www.merckmillipore.com/JP/ja/product/Anti-CTCF-Antibody,MM_NF-07-729

Pol2 , Pol2ser2 and H3K27ac:
https://www.nature.com/articles/nature13714
https://www.jstage.jst.go.jp/article/csf/33/1/33_07035/_article
CBP: https://www.citeab.com/antibodies/525781-606402-purified-anti-cbp-antibody?des=8d6fdfbea345bc59
P300: https://www.scbt.com/p/p300-antibody-c-20?requestFrom=search
Med1: https://www.scbt.com/p/trap220-antibody-c-19?requestFrom=search

Eukaryotic cell lines

Policy information about cell lines

Cell line source(s)	MCF-7 cells, RPE cells, 293T cells and Hela cells were from JCRB Cell Bank. B-cells and Fibroblast were obtained from the Children's Hospital of Philadelphia (Dr. Kosuke Izumi), which were reported in our previous study PMID: 22885700 and PMID: 25730767.
Authentication	None of the cell lines used were authenticated.
Mycoplasma contamination	All the cell lines tested negative for mycoplasm contamination.
Commonly misidentified lines (See ICLAC register)	No commonly misidentified cell lines were used.

ChIP-seq

Data deposition

☒ Confirm that both raw and final processed data have been deposited in a public database such as GEO.

☒ Confirm that you have deposited or provided access to graph files (e.g. BED files) for the called peaks.

Data access links
May remain private before publication.

The raw sequencing data and processed files are available at the GEO under the accession numbers GSE177045 (reviewer token sdchywuaxfcxdqf) https://www.ncbi.nlm.nih.gov/geo/query/acc.cgi?acc=GSE177045

Files in database submission

MCF7_CBP_Ctrl.fastq.gz
MCF7_CBP_E2.fastq.gz
MCF7_AFF4_Ctrl.fastq.gz
MCF7_AFF4_E2.fastq.gz
MCF7_CTCF_Ctrl.fastq.gz
MCF7_CTCF_E2.fastq.gz
MCF7_Mau2_Ctrl.fastq.gz
MCF7_Mau2_E2.fastq.gz
MCF7_Pol2_Ctrl.fastq.gz
MCF7_Pol2_E2_30min.fastq.gz
MCF7_Pol2_E2_45min.fastq.gz
MCF7_Pol2ser2_Ctrl.fastq.gz
MCF7_Pol2ser2_E2.fastq.gz
MCF7_Rad21_Ctrl.fastq.gz
MCF7_Rad21_E2_30min.fastq.gz
MCF7_Rad21_E2_45min.fastq.gz
MCF7_TAF1_Ctrl.fastq.gz
MCF7_TAF1_E2.fastq.gz
MCF7_p300_Ctrl.fastq.gz
MCF7_p300_E2.fastq.gz
MCF7_Input_Ctrl.fastq.gz
MCF7_Input_E2_30min.fastq.gz
MCF7_Input_E2_45min.fastq.gz
MCF7_Mau2_KD_Ctrl.fastq.gz
MCF7_Mau2_KD_E2.fastq.gz
MCF7_p300_KD_Ctrl.fastq.gz
MCF7_p300_KD_E2.fastq.gz
MCF7_Pol2_KD_Ctrl.fastq.gz
MCF7_Pol2_KD_E2.fastq.gz
MCF7_Pol2ser2_KD_Ctrl.fastq.gz
MCF7_Pol2ser2_KD_E2.fastq.gz
MCF7_CBP_KD_Ctrl.fastq.gz
MCF7_CBP_KD_E2.fastq.gz
MCF7_ER_Ctrl.fastq.gz
MCF7_ER_E2.fastq.gz
293T_Rad21_WT.fastq.gz
Bcell_Rad21_WT.fastq.gz
Fibroblast_Rad21_WT.fastq.gz
RPE_Rad21_WT.fastq.gz
HeLa_Rad21_WT_rep1.fastq.gz
HeLa_Rad21_WT_rep2.fastq.gz

RPE_Rad21_FBS-.fastq.gz
RPE_Rad21_FBS+.fastq.gz
RPE_Rad21_DRB-.fastq.gz
RPE_Rad21_DRB+.fastq.gz
RPE_Rad21_FBS+DRB.fastq.gz
RPE_SA1_FBS-.fastq.gz
RPE_SA1_FBS+.fastq.gz
RPE_CTCF.fastq.gz
RPE_Pol2_FBS-.fastq.gz
RPE_Pol2_FBS+.fastq.gz
RPE_Pol2ser2_FBS-.fastq.gz
RPE_Pol2ser2_FBS+.fastq.gz
RPE_Mau2_FBS-.fastq.gz
RPE_Mau2_FBS+.fastq.gz
RPE_Med1_FBS-.fastq.gz
RPE_Med1_FBS+.fastq.gz
RPE_K27ac.fastq.gz
RPE_Input_DRB-.fastq.gz
RPE_Input_DRB+.fastq.gz
RPE_Input_FBS-.fastq.gz
RPE_Input_FBS+DRB.fastq.gz
RPE_Input_FBS+.fastq.gz
Fibroblast_AFF4_CdLS.fastq.gz
Fibroblast_AFF4_CHOPS.fastq.gz
Fibroblast_AFF4_Normal.fastq.gz
Fibroblast_H3K27ac_CdLS.fastq.gz
Fibroblast_H3K27ac_CHOPS.fastq.gz
Fibroblast_H3K27ac_Normal.fastq.gz
Fibroblast_NIPBL_CdLS.fastq.gz
Fibroblast_NIPBL_CHOPS.fastq.gz
Fibroblast_NIPBL_Normal.fastq.gz
Fibroblast_Pol2_CdLS.fastq.gz
Fibroblast_Pol2_CHOPS.fastq.gz
Fibroblast_Pol2_Normal.fastq.gz
Fibroblast_Pol2ser5_CdLS.fastq.gz
Fibroblast_Pol2ser5_CHOPS.fastq.gz
Fibroblast_Pol2ser5_Normal.fastq.gz
Fibroblast_Rad21_CdLS.fastq.gz
Fibroblast_Rad21_CHOPS.fastq.gz
Fibroblast_Rad21_Normal.fastq.gz
Fibroblast_Input_CdLS.fastq.gz
Fibroblast_Input_CHOPS.fastq.gz
Fibroblast_Input_Normal.fastq.gz
RNAseq_MCF7_KD_Ctrl_rep1.fastq.gz
RNAseq_MCF7_KD_Ctrl_rep2.fastq.gz
RNAseq_MCF7_KD_E2_rep1.fastq.gz
RNAseq_MCF7_KD_E2_rep2.fastq.gz
RNAseq_MCF7_WT_Ctrl_rep1.fastq.gz
RNAseq_MCF7_WT_Ctrl_rep2.fastq.gz
RNAseq_MCF7_WT_E2_rep1.fastq.gz
RNAseq_MCF7_WT_E2_rep2.fastq.gz
RNAseq_CHOP_Fibroblast_rep1.fastq.gz
RNAseq_CHOP_Fibroblast_rep2.fastq.gz
RNAseq_CdLS_Fibroblast_rep1.fastq.gz
RNAseq_CdLS_Fibroblast_rep2.fastq.gz
RNAseq_Normal_Fibroblast_rep1.fastq.gz
RNAseq_Normal_Fibroblast_rep2.fastq.gz
293T_Rad21_WT.bed
Bcell_Rad21_WT.bed
Fibroblast_AFF4_CHOPS.bed
Fibroblast_AFF4_CdLS.bed
Fibroblast_AFF4_Normal.bed
Fibroblast_H3K27ac_CHOPS.bed
Fibroblast_H3K27ac_CdLS.bed
Fibroblast_H3K27ac_Normal.bed
Fibroblast_NIPBL_CHOPS.bed
Fibroblast_NIPBL_CdLS.bed
Fibroblast_NIPBL_Normal.bed
Fibroblast_Pol2_CHOPS.bed
Fibroblast_Pol2_CdLS.bed
Fibroblast_Pol2_Normal.bed
Fibroblast_Pol2ser5_CHOPS.bed
Fibroblast_Pol2ser5_CdLS.bed
Fibroblast_Pol2ser5_Normal.bed
Fibroblast_Rad21_CHOPS.bed
Fibroblast_Rad21_CdLS.bed

Fibroblast_Rad21_Normal.bed
Fibroblast_Rad21_WT.bed
HeLa_Rad21_WT_rep1.bed
HeLa_Rad21_WT_rep2.bed
MCF7_AFF4_Ctrl.bed
MCF7_AFF4_E2.bed
MCF7_CBP_Ctrl.bed
MCF7_CBP_E2.bed
MCF7_CBP_KD_Ctrl.bed
MCF7_CBP_KD_E2.bed
MCF7_CTCF_Ctrl.bed
MCF7_CTCF_E2.bed
MCF7_ER_Ctrl.bed
MCF7_ER_E2.bed
MCF7_Mau2_Ctrl.bed
MCF7_Mau2_E2.bed
MCF7_Mau2_KD_Ctrl.bed
MCF7_Mau2_KD_E2.bed
MCF7_Pol2_Ctrl.bed
MCF7_Pol2_E2_30min.bed
MCF7_Pol2_E2_45min.bed
MCF7_Pol2_KD_Ctrl.bed
MCF7_Pol2_KD_E2.bed
MCF7_Pol2ser2_Ctrl.bed
MCF7_Pol2ser2_E2.bed
MCF7_Pol2ser2_KD_Ctrl.bed
MCF7_Pol2ser2_KD_E2.bed
MCF7_Rad21_Ctrl.bed
MCF7_Rad21_E2_30min.bed
MCF7_Rad21_E2_45min.bed
MCF7_TAF1_Ctrl.bed
MCF7_TAF1_E2.bed
MCF7_p300_Ctrl.bed
MCF7_p300_E2.bed
MCF7_p300_KD_Ctrl.bed
MCF7_p300_KD_E2.bed
RPE_CTCF.bed
RPE_K27ac.bed
RPE_Mau2_FBS+.bed
RPE_Mau2_FBS-.bed
RPE_Med1_FBS+.bed
RPE_Med1_FBS-.bed
RPE_Pol2_FBS+.bed
RPE_Pol2_FBS-.bed
RPE_Pol2ser2_FBS+.bed
RPE_Pol2ser2_FBS-.bed
RPE_Rad21_DRB+.bed
RPE_Rad21_DRB-.bed
RPE_Rad21_FBS+.bed
RPE_Rad21_FBS+DRB.bed
RPE_Rad21_FBS-.bed
RPE_Rad21_WT.bed
RPE_SA1_FBS+.bed
RPE_SA1_FBS-.bed
RNAseq_MCF7_KD_Ctrl_rep1.count.txt
RNAseq_MCF7_KD_Ctrl_rep2.count.txt
RNAseq_MCF7_KD_E2_rep1.count.txt
RNAseq_MCF7_KD_E2_rep2.count.txt
RNAseq_MCF7_WT_Ctrl_rep1.count.txt
RNAseq_MCF7_WT_Ctrl_rep2.count.txt
RNAseq_MCF7_WT_E2_rep1.count.txt
RNAseq_MCF7_WT_E2_rep2.count.txt
RNAseq_CHOP_Fibroblast_rep1.count.txt
RNAseq_CHOP_Fibroblast_rep2.count.txt
RNAseq_CdLS_Fibroblast_rep1.count.txt
RNAseq_CdLS_Fibroblast_rep2.count.txt
RNAseq_Normal_Fibroblast_rep1.count.txt
RNAseq_Normal_Fibroblast_rep2.count.txt
New_MCF7_Pol2_NIPBLKD_Ctrl
New_MCF7_Pol2_NIPBLKD_E2
New_MCF7_Pol2_Rad21KD_Ctrl
New_MCF7_Pol2_Rad21KD_E2
New_MCF7_Pol2_WT_Ctrl
New_MCF7_Pol2_WT_E2
New_MCF7_Pol2ser2_NIPBLKD_Ctrl
New_MCF7_Pol2ser2_NIPBLKD_E2

	New_MCF7_Pol2ser2_Rad21KD_Ctrl<br>New_MCF7_Pol2ser2_Rad21KD_E2<br>New_MCF7_Pol2ser2_WT_Ctrl<br>New_MCF7_Pol2ser2_WT_E2<br>New_RPE_Rad21_FBS+<br>New_RPE_Rad21_FBS-<br>New_MCF7_Input_Rad21KD_Ctrl<br>New_MCF7_Input_Rad21KD_E2<br>New_MCF7_Input_WT_Ctrl<br>New_MCF7_Input_WT_E2
Genome browser session<br>(e.g. UCSC)	The peak file (.bed) are provided in the GEO submission. These files can be imported directly into WashU Browser or UCSC genome browser.

Methodology

Replicates	RNA-seq was performed in duplicates. ChIP-seq was performed with time course treatment of E2. ChIP-seq was also confirmed by public data as described in manuscript.
Sequencing depth	The statistics and quality check results are completely listed in Supplementary Table 1.
Antibodies	Rabbit polyclonal antibody for Rad21 has been described in PMID:26051894. Antibody for Pol2 (#14958) was from Cell Signaling Technology. Antibodies for Mau2 (ab46906) and SA1 (ab4457) were from Abcam. Antibodies for TAF (A303-505A) and AFF4 (A302-538A) were from Bethyl Laboratory. CTCF (07-729) antibody was from Merck Millipore. Antibodies for Pol2ser2 and H3K27ac were kindly provided by Dr. H Kimura (TITech). Antibodies for P300 (sc-585) and Med1 (sc-5334) were from Santa Cruz Biotechnology.Antibody for CBP (606402) was from BioLegend.
Peak calling parameters	Read mapping: Bowtie41 version 1.2.2 with "-n2 -m1" parameters; hg38 reference genome.<br>Peak calling: MACS under the default parameters, input files as control; hg38 reference genome.
Data quality	Quality check by FastQC and SSP, and the quality check results are summarized in Supplementary Table 1.
Software	Bowtie version 1.2.2; DROMPA version 3.7.2; MACS2 version 2.2.6; Bedtools version 2.29.2; Samtools version 1.9;
